# Fast myocardial *T*_1ρ_ mapping in mice using k-space weighted image contrast and a Bloch simulation-optimized radial sampling pattern

**DOI:** 10.1007/s10334-021-00951-y

**Published:** 2021-09-07

**Authors:** Maximilian Gram, Daniel Gensler, Patrick Winter, Michael Seethaler, Paula Anahi Arias-Loza, Johannes Oberberger, Peter Michael Jakob, Peter Nordbeck

**Affiliations:** 1 Department of Internal Medicine I, University Hospital Würzburg, Oberdürrbacher Str. 6, 97080 Würzburg, Germany; 2 Experimental Physics 5, University of Würzburg, Würzburg, Germany; 3 Comprehensive Heart Failure Center (CHFC), University Hospital Würzburg, Würzburg, Germany; 4 Department of Nuclear Medicine, University Hospital Würzburg, Würzburg, Germany

**Keywords:** *T*_1rho_, *T*_1ρ_ mapping, *T*_1ρ_ dispersion, Spin lock, Radial, KWIC, Cardiac, Small animal, Mice

## Abstract

**Purpose:**

*T*_1ρ_ dispersion quantification can potentially be used as a cardiac magnetic resonance index for sensitive detection of myocardial fibrosis without the need of contrast agents. However, dispersion quantification is still a major challenge, because *T*_1ρ_ mapping for different spin lock amplitudes is a very time consuming process. This study aims to develop a fast and accurate *T*_1ρ_ mapping sequence, which paves the way to cardiac *T*_1ρ_ dispersion quantification within the limited measurement time of an in vivo study in small animals.

**Methods:**

A radial spin lock sequence was developed using a Bloch simulation-optimized sampling pattern and a view-sharing method for image reconstruction. For validation, phantom measurements with a conventional sampling pattern and a gold standard sequence were compared to examine *T*_1ρ_ quantification accuracy. The in vivo validation of *T*_1ρ_ mapping was performed in *N* = 10 mice and in a reproduction study in a single animal, in which ten maps were acquired in direct succession. Finally, the feasibility of myocardial dispersion quantification was tested in one animal.

**Results:**

The Bloch simulation-based sampling shows considerably higher image quality as well as improved *T*_1ρ_ quantification accuracy (+ 56%) and precision (+ 49%) compared to conventional sampling. Compared to the gold standard sequence, a mean deviation of − 0.46 ± 1.84% was observed. The in vivo measurements proved high reproducibility of myocardial *T*_1ρ_ mapping. The mean *T*_1ρ_ in the left ventricle was 39.5 ± 1.2 ms for different animals and the maximum deviation was 2.1% in the successive measurements. The myocardial *T*_1ρ_ dispersion slope, which was measured for the first time in one animal, could be determined to be 4.76 ± 0.23 ms/kHz.

**Conclusion:**

This new and fast *T*_1ρ_ quantification technique enables high-resolution myocardial *T*_1ρ_ mapping and even dispersion quantification within the limited time of an in vivo study and could, therefore, be a reliable tool for improved tissue characterization.

**Supplementary Information:**

The online version contains supplementary material available at 10.1007/s10334-021-00951-y.

## Introduction

Cardiac magnetic resonance imaging (cMRI) has become an increasingly important imaging technique, which enables various non-invasive diagnostic options in clinical cardiology and basic cardiologic research. In addition to informative morphological and functional investigations, cMRI offers the possibility of enhanced tissue characterization to investigate tissue defects like edema, inflammation or fibrosis [1–3]. There are several methods facilitating magnetic resonance imaging to enable tissue characterization, such as late gadolinium enhancement (LGE), *T*_1_- and *T*_2_ mapping, and the quantification of the extracellular volume [4–8]. However, some of these methods require the administration of gadolinium contrast agent, which is contraindicated in certain patients, especially those with reduced renal function.

Native *T*_1ρ_ quantification has been emerged to be a promising alternative. The *T*_1ρ_ relaxation mechanism, also called spin lattice relaxation in the rotating frame, uses an on-resonant radiofrequency pulse that locks the magnetization and prohibits free relaxation of the spin ensemble [9–11]. This applied spin lock (SL) pulse causes a high sensitivity to low frequency processes at the molecular and cellular level (e.g. of biologic macromolecules) [12–14]. In the field of cMRI, the SL method, using a moderate locking amplitude, has been shown to suppress low frequency relaxation mechanisms that obscure endogenous contrast [15]. In chronic infarcts in the swine model, *T*_1ρ_ revealed a significant increase compared to healthy remote myocardium even without the use of contrast agents [15]. Compared to other native quantification techniques, *T*_1ρ_ improves the contrast ratio between diseased and healthy myocardial tissue [16–18]. In experimental models as well as in vivo, *T*_1ρ_ has already been shown to be a sensitive marker for the detection of several tissue damages like edema, ischemia, fibrosis and myocardial infarction [19–22]. Moreover, *T*_1ρ_ gives the possibility for dispersion measurements by manipulation of the effective SL pulse amplitude, enabling additional contrast mechanisms and further improved tissue characterization [11]. In this context it could already be shown that *T*_1ρ_ dispersion quantification can be used as a myocardial biomarker for the classification of different stages of fibrosis [19]. This dispersion behavior is of great interest and might be highly beneficial not only in basic research in various animal models, but also in various scenarios in clinical practice [23–25]. However, fast and accurate *T*_1ρ_ dispersion quantification in cMRI is still a major imaging challenge, since the preparation of *T*_1ρ_ must be carried out immediately before each acquisition [26, 27] and the acquisition time is strongly limited by physiological parameters such as the breathing cycle and heart rate. For the calculation of a single *T*_1ρ_ map, several images with different *T*_1ρ_ weightings must be acquired. To quantify the dispersion, several maps must be measured using different SL amplitudes. This procedure can lead to excessive measurement times, especially in small animal studies.

Despite the many promising possibilities of cardiac *T*_1ρ_ dispersion quantification, only minor attention has been paid to the technique itself so far. To date, there are only a few publications that deal with myocardial *T*_1ρ_ dispersion analysis. In the work presented by Witschey et al. [15] myocardial *T*_1ρ_ mapping was performed in vivo using an infarction model in pigs. Here, the infarction area could be clearly visualized using cardiac *T*_1ρ_ mapping. However, *T*_1ρ_ dispersion measurements were only performed ex vivo, revealing a significant difference in the dispersion behavior between infarcted, border zone and remote myocardium. Musthafa et al. [20] presented a study in a mouse infarction model at which *T*_1ρ_ mapping was performed at several time points after infarction, accounting a significant increase of *T*_1ρ_ at day 7 after infarction. The sequence used for high-resolution *T*_1ρ_ mapping was based on respiratory and heartbeat triggered spin locking followed by a single Cartesian spin echo readout. This acquisition procedure ensured a high signal-to-noise ratio (SNR) but was relatively slow, requiring a total scan time of approximately 20 min for a single-slice T_1ρ_ map, although only four different *T*_1ρ_ weighted images were acquired for mapping. This work also introduced an accelerated proof of concept *T*_1ρ_ dispersion measurement with reduced spatial resolution. However, these measurements only seem to roughly estimate the dispersion behavior and do not allow an exact pixel-by-pixel quantification. In the work published by Yin et al. [19] *T*_1ρ_ dispersion analysis has been performed in a fibrosis model in several dogs in vivo. The authors introduced a dispersion dependent myocardial biomarker that enables the distinction between different grades of fibrosis. A drawback of the study is that only two relaxation maps were used for dispersion quantification and one of these maps did not represent a true *T*_1ρ_ map but rather a *T*_2_ map. Over all, the main reason why there is no suitable myocardial *T*_1ρ_ dispersion quantification method so far is the fact that an accurate measurement can take several hours.

The aim of our work is to introduce a very fast and accurate *T*_1ρ_ quantification technique that enables high-resolution myocardial *T*_1ρ_ mapping and *T*_1ρ_ dispersion quantification even within the limited time window of a small animal in vivo study. For this, a Bloch simulation-optimized imaging sequence using high flip angles and a radial view-sharing method has been developed ensuring high SNR and efficient data sampling. In our study, the new radial sampling technique was compared in phantom measurements with a conventional radial acquisition scheme and a reference gold standard technique. In addition, measurements were carried out in *N* = 10 healthy mice to test the in vivo applicability and to carry out, for the first time, quantitative dispersion imaging in the myocardium.

## Methods

### Sequence design

**Fig. 1:**
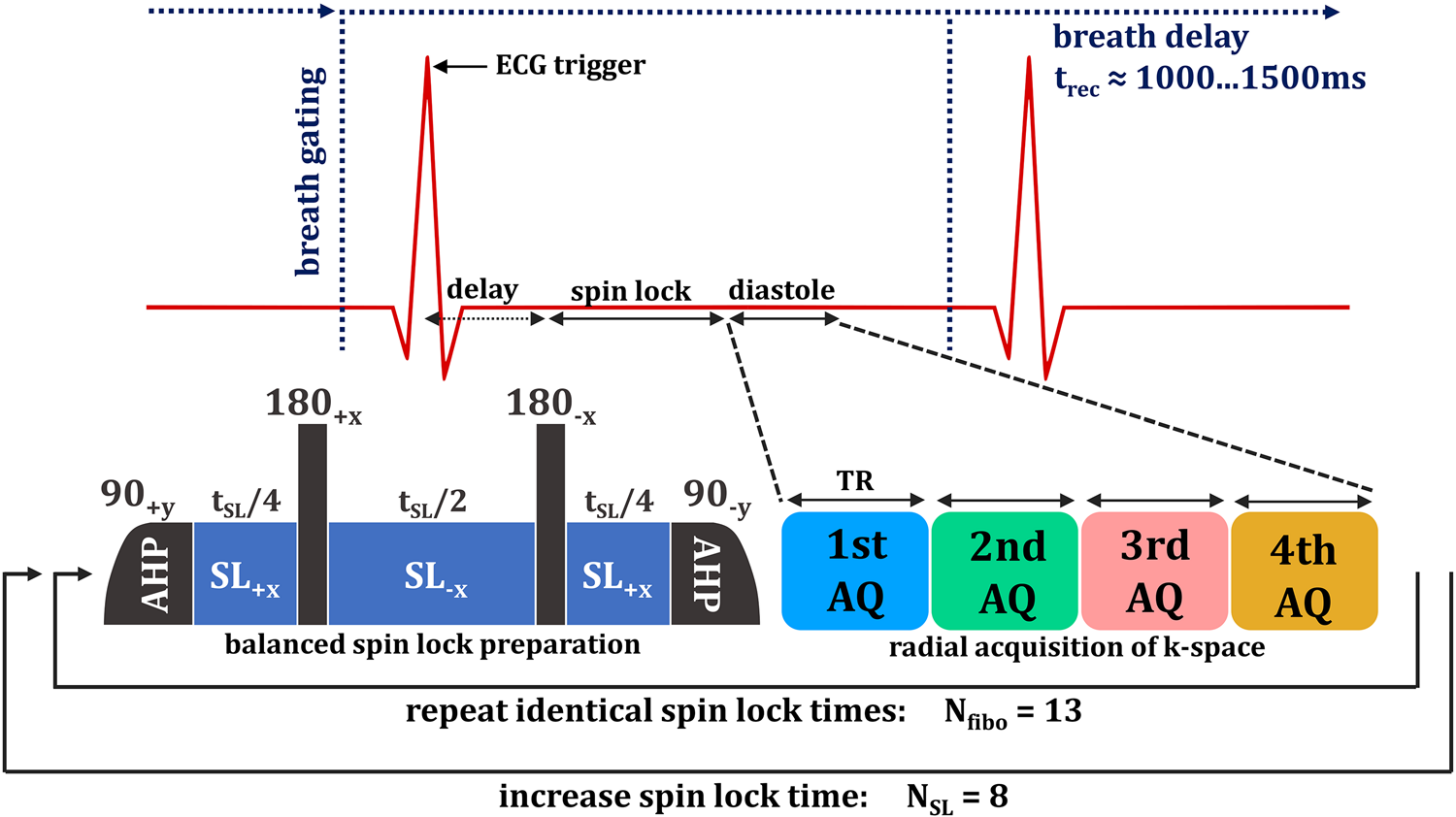
Sequence design for myocardial *T*_1ρ_ mapping in mice. After prospective respiratory gating a trigger on the R-wave has been used followed by a dynamic trigger delay and a balanced SL preparation module [28]. Thereafter, four radial spokes of the k-space were acquired in end diastole during one heartbeat. This procedure was repeated in each respiratory cycle separated by a waiting time *t*_rec_ for magnetization recovery. For each *T*_1ρ_ weighting, 13 repetitions were used

The optimized *T*_1ρ_ mapping sequence has been designed to acquire a series of *T*_1ρ_ weighted images with different SL times *t*_SL_ within a single measurement. Therefore, a signal intensity dependent radial sampling pattern and a k-space view-sharing method has been used. *T*_1ρ_ preparation was performed by a balanced spin locking module, which includes two adiabatic half-passage (AHP) excitation pulses, three continuous wave SL pulses using alternating phases and two opposite 180° refocusing pulses for improved *B*_0_ and *B*_1_ insensitivity [28, 29]. For data acquisition, a golden angle radial gradient echo readout was used [30], acquiring four radial spokes after each SL preparation (Fig. 1). The acquisition window was positioned in end diastole using a dynamic trigger delay (depending on *t*_SL_) after the R-wave of the ECG signal. Each preparation experiment was separated by a waiting time *t*_rec_ for magnetization recovery, which is dependent on the respiratory cycle rate. Data sampling was segmented into 13 preparation experiments with identical preparation characteristics (identical SL time *t*_SL_ and SL amplitude *f*_SL_). These 13 identical preparation experiments were carried out with eight different SL times, enabling the reconstruction of eight images with different *T*_1ρ_ weightings using a k-space weighted image contrast (KWIC) filtered view-sharing method [31]. This leads to 13 × 8 = 104 consecutive preparation experiments and consequently 104 × 4 = 416 radial acquisitions in total for the generation of a single *T*_1ρ_ map. The acquisition time at a respiratory rate of 1 Hz is ≈1.7 min.

### Concept of Bloch sorting

The fundamental idea of our fast *T*_1ρ_ mapping technique is based on three key points. First, the increase of SNR using high flip angles in the gradient echo readouts. Second, the algorithmic search for an optimal radial sampling pattern based on Bloch simulations. Third, the image reconstruction of undersampled data using a KWIC-filtered view-sharing method.

Due to the very high heart rate in mice (≈ 450 bpm), only a few readouts after the spin lock preparation are possible in the acquisition window of the diastole (≈ 20 ms). Hence, short repetition times TR (≈ 5 ms) and only four readouts per preparation were used. The waiting time for magnetization recovery is set by the respiratory cycle (breath gating, *t*_rec_ ≈ 1500 ms) and is therefore relatively long. Since this means that only a small number of acquisitions are carried out over time, the signal level must be maximized. In this specific case, the maximum of the averaged signal intensity $\overline{S }$ is achieved if appropriately high flip angles are used in the RF pulse train. Without taking relaxation effects into account, the optimal flip angle can be calculated as follows:

1 $${\alpha }_{\mathrm{opt}}=\underset{\alpha }{\mathrm{arg max}} \left[\overline{S }\left(\alpha \right)\right]=\underset{\alpha }{\mathrm{arg max}}\left[{\sum }_{k=1}^{4}\mathrm{sin}\left(\alpha \right) {\mathrm{cos}\left(\alpha \right)}^{ k-1}\right]={43.51}^{^\circ }$$

**Fig. 2:**
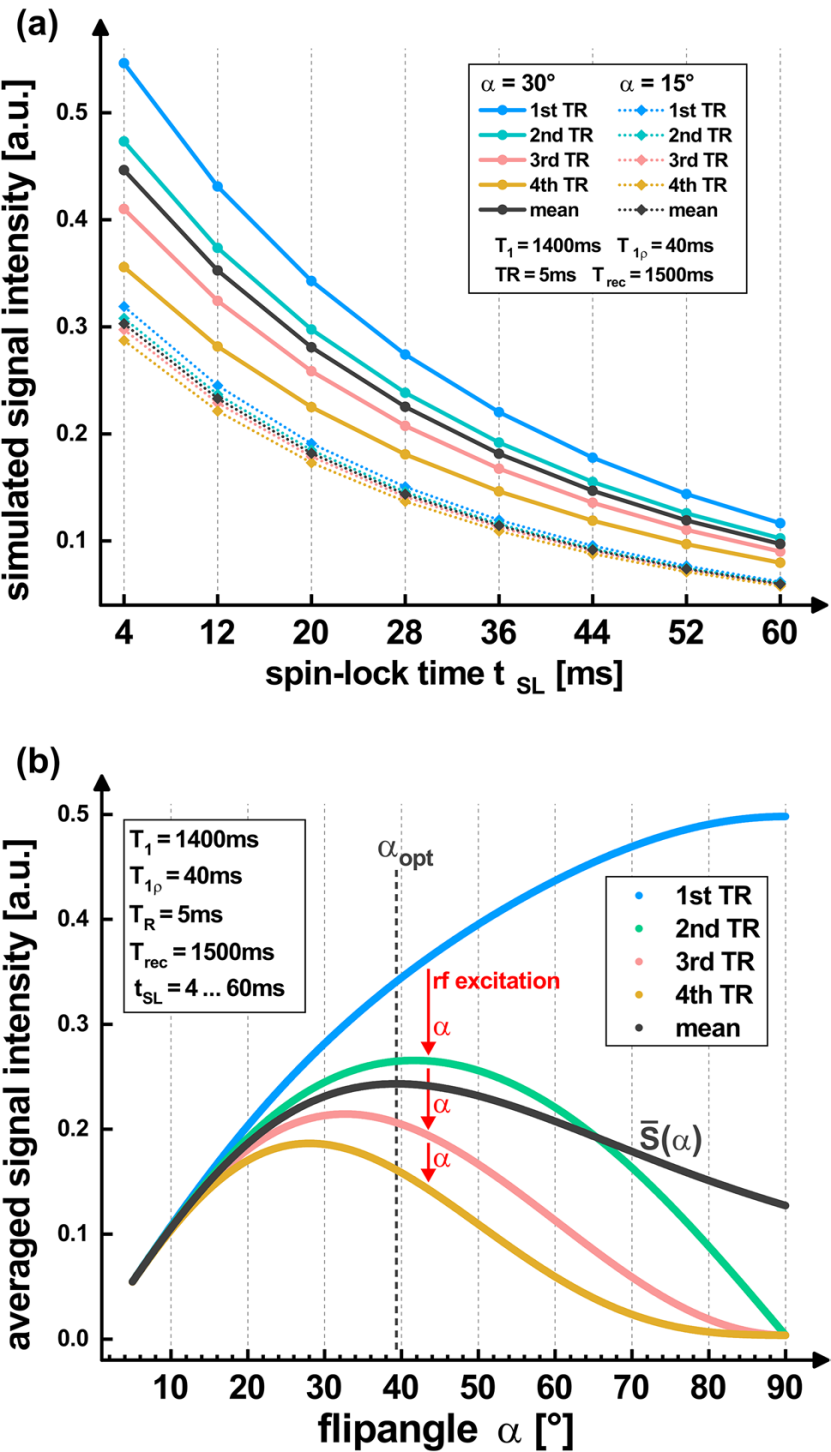
Optimization of the flip angle for the radial gradient echo readout. **a** Results of the Bloch simulation for two exemplary flip angles. At low flip angles, the signal intensities of the different readouts differ only slightly. At higher flip angles, a variation in the signal intensities is observed. **b** The plot shows the simulated signal averaged over all *t*_SL_ for different flip angles. For the sequence parameters TR = 5 ms, *t*_rec_ = 1500 ms, *t*_SL_ = 4…60 ms and the relaxation times *T*_1_ = 1400 ms and *T*_1ρ_ = 40 ms (typical for myocardial tissue), the mean signal level $\overline{S }$ is maximized for *α*_opt_ = 39.35°. This value strongly depends on the number of readouts after preparation (NR = 4)

By considering typical relaxation times of myocardial tissue (*T*_1ρ_ = 40 ms [20], *T*_1_ = 1400 ms [32]) and the sequence timings (TR = 5 ms, *t*_rec_ = 1500 ms) described above, the signal maximum is reached at a slightly different value. In Fig. 2a the signal of the NR = 4 readouts was simulated for different SL times (*t*_SL_ = 4, 12, 20, 28, 36, 44, 52, 60 ms) by solving the Bloch equations [33]. In Fig. 2b, the simulation was carried out for different flip angles and plotted averaged over all SL times and readouts. The signal maximum is reached at *α*_opt_ = 39.35°. Based on this estimate, we consistently used *α* = 40° for all measurements and simulations in this study. Since the *T*_1_ recovery is not complete between sequence repetitions, we carried out two dummy cycles prior to the first preparation experiment to prevent increased signals in the first acquisitions. The high flip angles are advantageous here because the steady-state is reached quickly.

**Fig. 3:**
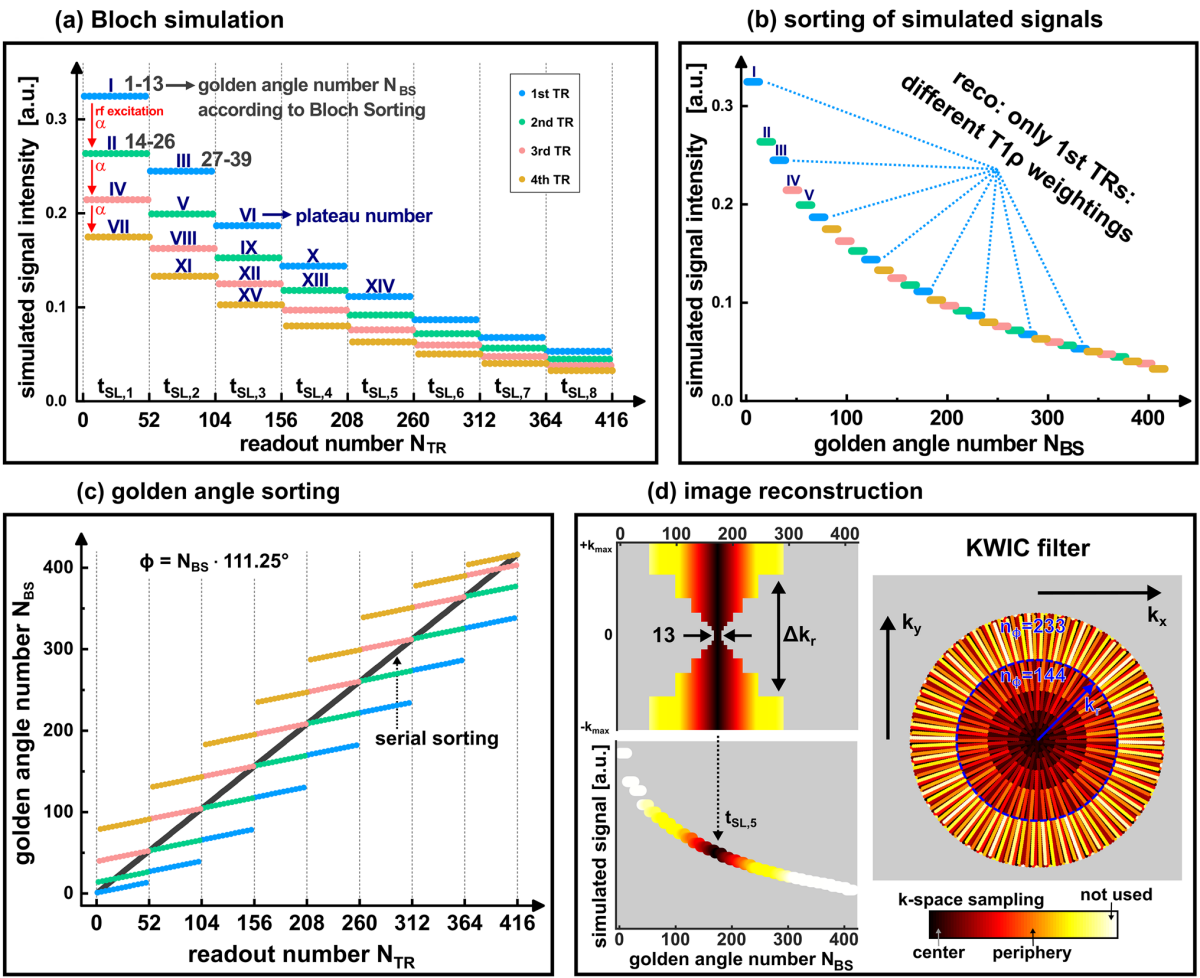
Concept of Bloch sorting (**a**–**c**) and the image reconstruction technique (**d**). **a** The expected signal intensity is plotted versus the chronological readout number of the acquired spokes. For Bloch simulation the values *T*_1_ = 1400 ms, *T*_1ρ_ = 60 ms, TR = 5 ms, *t*_rec_ = 1000 ms, *α* = 40° and *t*_SL_ = 4…102 ms (linear spacing) were used in the phantom experiments. The colors of the plateaus represent the different acquisition windows after SL preparation (see Figs. 1 and 2). The roman numerals indicate the sorting position of the plateaus. The numbers in gray represent the indices of the golden angles numbers. **b** Signal intensity of subsequent golden angles after Bloch sorting. In the ideal case, the curve is monotonically decreasing. The change in contrast between subsequent spokes is minimized. **c** Bloch simulation-optimized sampling pattern. A projection angle *ϕ* is calculated for each readout by linking subsequent golden angles to the sorting position. **d** KWIC profile for the reconstruction of a *T*_1ρ_ weighted image and the corresponding k-space sampling trajectory. The 13 readouts directly after *T*_1ρ_ preparation are used exclusively to sample the corresponding k-space center. The k-space periphery is also filled with other weightings

The signal maximization generated using high flip angles for the readout (*α* = 40°) has the disadvantage of strong signal variations after each *T*_1ρ_ preparation (Fig. 3a). This would ultimately lead to incorrect *T*_1ρ_ weighting if all projections were used equally for the sampling of k-space. A fundamental idea of our method is therefore to generate a smooth variation of signal intensities across k-space using an optimized sorting of golden angles. For this a Bloch simulation-optimized sampling scheme was developed. The signal intensity for every acquisition window was roughly predicted prior to the measurement using the known sequence parameters (TR, *t*_rec_ and *α*) and estimated *T*_1_ and *T*_1ρ_ values of the probe under investigation by solving the Bloch equations [33]. The results were used to create a sorting (Bloch sorting) of the predicted signal levels for the corresponding acquisition windows (Fig. 3b). The projection angle $\phi$ was calculated for each radial readout by linking neighboring golden angles (111.25°, 222.50°, 333.75°, …) with the corresponding Bloch sorting position ${N}_{\mathrm{BS}}$ (Fig. 3c).

2 $$\phi ={N}_{\mathrm{BS}}\cdot \frac{360^\circ }{1+\sqrt{5}}\approx {N}_{\mathrm{BS}}\cdot 111.25^\circ$$

The image reconstruction was performed using an adapted KWIC-filtered technique [31] (Fig. 3d). Here, the k-space center for a desired *T*_1ρ_ weighting (with desired *t*_SL_) is exclusively selected from the first readout after preparation. This ensures that the main image contrast of the specific *T*_1ρ_ weighted image is provided by the correct SL time. Since we have 13 (Fibonacci number) radial projections available for each preparation experiment, the sampling density in the azimuthal direction is homogeneous and only two different azimuthal gaps exist (Fig. 3d) [30]. Due to the Bloch sorting technique, the change in contrast between neighboring golden angles used for the k-space filling (based on the KWIC filter) is minimized for the k-space center. The k-space peripherals were also chosen from other acquisition windows as well as other T_1ρ_ weightings. Here, the k-space was filled in segments according to the Bloch sorting principle. In each segment, the total number of projections ${n}_{\phi }$ agrees to a Fibonacci number (13, 21, 34, 55, …) to ensure a homogenous sampling density. We further ensured that the Nyquist criterion was met in each region of the segments (detailed explanation in the supplementary material, Online Fig. 1). For ${n}_{\phi }$ projections in a segment, the number of samples in radial direction ${n}_{r}$ (*k*_r_ direction, Fig. 3d) was linked to the condition ${n}_{\phi }>\pi \cdot { n}_{\mathrm{r}}$. The reconstruction of the radial k-space data was done using a nonuniform fast Fourier transform (NUFFT) with an open source software toolbox [34, 35].

### Phantom measurements

All measurements were performed on a 7.0 T small animal imaging system (Bruker BioSpec 70/30, Bruker BioSpin MRI GmbH, Ettlingen, Germany) with a maximum gradient field strength of 470 mT/m. A 35 mm homebuilt quadrature transmit-receive birdcage was used for signal detection. The phantom used consisted of four cylindrical sample tubes with a diameter of 17 mm and a length of 120 mm. The tubes were filled with different concentrations (10, 15, 20, and 25%) of BSA (Bovine Serum Albumin, Sigma-Aldrich, St. Louis, MO, USA) resulting in different *T*_1ρ_ values in the typical range of biological tissue. The sample tubes were arranged in a quadratic array, which was placed in the isocenter of the magnet. In the phantom measurements, ECG triggering of the sequence was deactivated and the recovery time was fixed to a constant value *t*_rec_ = 5000 ms.

To demonstrate the advantages of the Bloch simulation-based sampling scheme, the image quality and the T_1ρ_ quantification accuracy were compared with a conventional sampling scheme using a serial sorting of golden angles for subsequent readouts (Fig. 3c). In this setup, the projection angles were not optimized for the expected signal intensity. The remaining sequence parameters for the acquired *T*_1ρ_ weighted images were adjusted identical for both sampling schemes: field of view (FOV) = 38.4 × 38.4 mm^2^, slice thickness = 1.5 mm, acquired/reconstructed resolution = 128 × 128 pixels, repetition time (TR) = 5.0 ms, echo time (TE) = 2.0 ms, bandwidth = 75 kHz, flip angle *α* = 40°, acquired spokes after SL preparation = 4, *t*_SL_ = 4…102 ms (8 different, linear spacing), *f*_SL_ = 1500 Hz. The calculation of a single *T*_1ρ_ map required 104 (13 × 8) SL preparation experiments.

An artifact/SNR analysis was performed to determine the sampling scheme that achieves the best image quality with least artifacts, due to the undersampling of the k-space and the KWIC filter based image reconstruction. Therefore, the SNR for every *T*_1ρ_ weighted image has been calculated and averaged over all reconstructed images. The individual SNR values were calculated from the magnitude images using four signal masks and a noise mask (Fig. 5) avoiding the edges of the phantoms. The ratio was built from the mean value within the signal masks and the standard deviation within the noise mask. In addition, the coefficient of determination *R*^2^, which represents a measure of the agreement with the mono-exponential model function, has been calculated pixel-wise for each fitting process of the *T*_1ρ_ exponential decay and was finally averaged to obtain a global *R*^2^ indicator for both sampling schemes.

For validation of the *T*_1ρ_ quantification accuracy pixel-wise comparisons with a Cartesian SL prepared turbo spin echo (TSE) sequence were performed, which serves as the gold standard reference. The TSE sequence parameters were chosen similar to the radial sequence and the identical balanced spin lock preparation was used: FOV = 38.4 × 38.4 mm^2^, slice thickness = 1.5 mm, resolution = 128 × 128 pixels, TR = 5031.4 ms (including *t*_rec_), TE = 7.0 ms, bandwidth = 50 kHz, turbo factor = 4, *t*_rec_ = 5000 ms, *t*_SL_ = 4…102 ms (eight different, linear spacing), *f*_SL_ = 1500 Hz. *T*_1ρ_ mapping using the reference TSE sequence required 256 (32 × 8) SL preparation experiments. Hence, our new radial *T*_1ρ_ mapping sequence is about 2.5 times (32:13) faster than the TSE reference measurement.

Furthermore, the accuracy of the *T*_1ρ_ dispersion quantification was examined by comparing the novel radial Bloch sorting technique and the TSE reference. Here, *T*_1ρ_ mapping was performed for eight different SL amplitudes (f_SL_ = 750…2500 Hz, linear spacing). Thus 8 × 8 × 13 = 832 preparation experiments were required for the radial acquisitions and 8 × 8 × 32 = 2048 preparations for the TSE sequence. For data analysis, circular regions of interest (ROIs) were drawn at the positions of the four *T*_1ρ_ sample probes. In these ROIs, the *T*_1ρ_ mapping results were compared pixel-wise with the corresponding values obtained from the TSE reference sequence. The mean quantification accuracy and its variance were determined based on the eight mapping experiments. In addition, the *T*_1ρ_ dispersion was analyzed using a linear dispersion model to the eight acquired *T*_1ρ_ maps with various SL amplitudes *f*_SL_.

3 $${T}_{1\uprho }\left({f}_{\mathrm{SL}}\right)={T}_{1\uprho }^{0}+{m}_{1\uprho }\cdot {f}_{\mathrm{SL}}$$

The quantification accuracy of the dispersion offset ${{T}_{1\uprho }\left({f}_{\mathrm{SL}}=0\right)=T}_{1\uprho }^{0}$ and the dispersion slope ${m}_{1\uprho }$ was also investigated by a pixel-wise examination for each sample tube, identifying its deviation from the corresponding pixel in the TSE reference.

### In vivo measurements

To check the applicability of our new method in vivo, measurements were carried out in healthy mice. Here the Bloch sorting scheme was used to quantify the *T*_1ρ_ relaxation time and *T*_1ρ_ dispersion, since—with respect of the results of the phantom measurements—the Serial sorting scheme did not allow a reasonable application in vivo.

The mice (Naval Medical Research Institute, Charles River Laboratories MA) were imaged in prone position. The animals were anesthetized with isoflurane inhalation (1.5–2 Vol. % in oxygen) and were kept at a constant body temperature of 37 °C. For signal detection the same 35 mm quadrature transmit-receive birdcage as for the phantom measurements was used. Two ECG electrodes attached to the forepaws of the mice were used for ECG triggering and a pressure sensitive balloon placed on the abdominal wall for breath gating. All experimental procedures were in accordance with institutional guidelines and were approved by local authorities.

Myocardial *T*_1ρ_ mapping was performed in *N* = 10 mice in a single slice for *f*_SL_ = 1500 Hz. After a standard planning procedure using high-resolution cine images, a midventricular short-axis imaging slice has been selected for the *T*_1ρ_ measurements. The acquisition window was positioned in end diastole using a variable trigger delay dependent on *t*_SL_. The recovery time *t*_rec_ was chosen dependent on the respiratory cycle, which was ≈ 1500 ms. The further sequence parameters were adjusted similar to the phantom measurements: FOV = 32 × 32 mm^2^, slice thickness = 1.5 mm, TR = 4.7 ms, TE = 1.9 ms, bandwidth = 75 kHz, *α* = 40°, acquired spokes after SL preparation = 4, acquired/reconstructed resolution = 128 × 128 pixels, *t*_SL_ = 4…60 ms (eight different, linear spacing). The in vivo measurement time for each *T*_1ρ_ map was approximately 2.5 min.

For analysis of the best case reproducibility of the radial *T*_1ρ_ mapping sequence, a series of ten *T*_1ρ_ maps with identical SL amplitudes was acquired in one animal in direct succession. A global left ventricular ROI has been selected and was then copied to every repeated *T*_1ρ_ map. Subsequently, the mean *T*_1ρ_ values as well as the standard deviations have been calculated at the position of the ROIs. In addition, a comparative measurement using an equivalent Cartesian gradient echo readout was carried out in one animal. Here, fully sampled data sets were acquired for all *T*_1ρ_ weighted images, leading to a total measurement time of ≈ 6.2 min for the matrix size of 128 × 128. All *T*_1ρ_ maps obtained were analyzed in a left ventricular segmentation model according to the American Heart Association (AHA).

Furthermore, a detailed analysis of the *T*_1ρ_ dispersion was carried out in one animal. Therefore, eight *T*_1ρ_ maps with different SL amplitudes *f*_SL_ = 750…2500 Hz (eight different, linear spacing, 8 × 8 × 13 = 832 preparation experiments) were acquired. With the acquired data, eight myocardial *T*_1ρ_ maps and a quantitative *T*_1ρ_ dispersion slope map were calculated (Eq. 3). Finally, the dispersion behavior of myocardial tissue and the left ventricular blood pool was quantitatively analyzed. Here the measurement time for the acquisition of the complete *T*_1ρ_ dispersion map was approximately 20 min.

## Results

### Phantom measurements

**Fig. 4:**
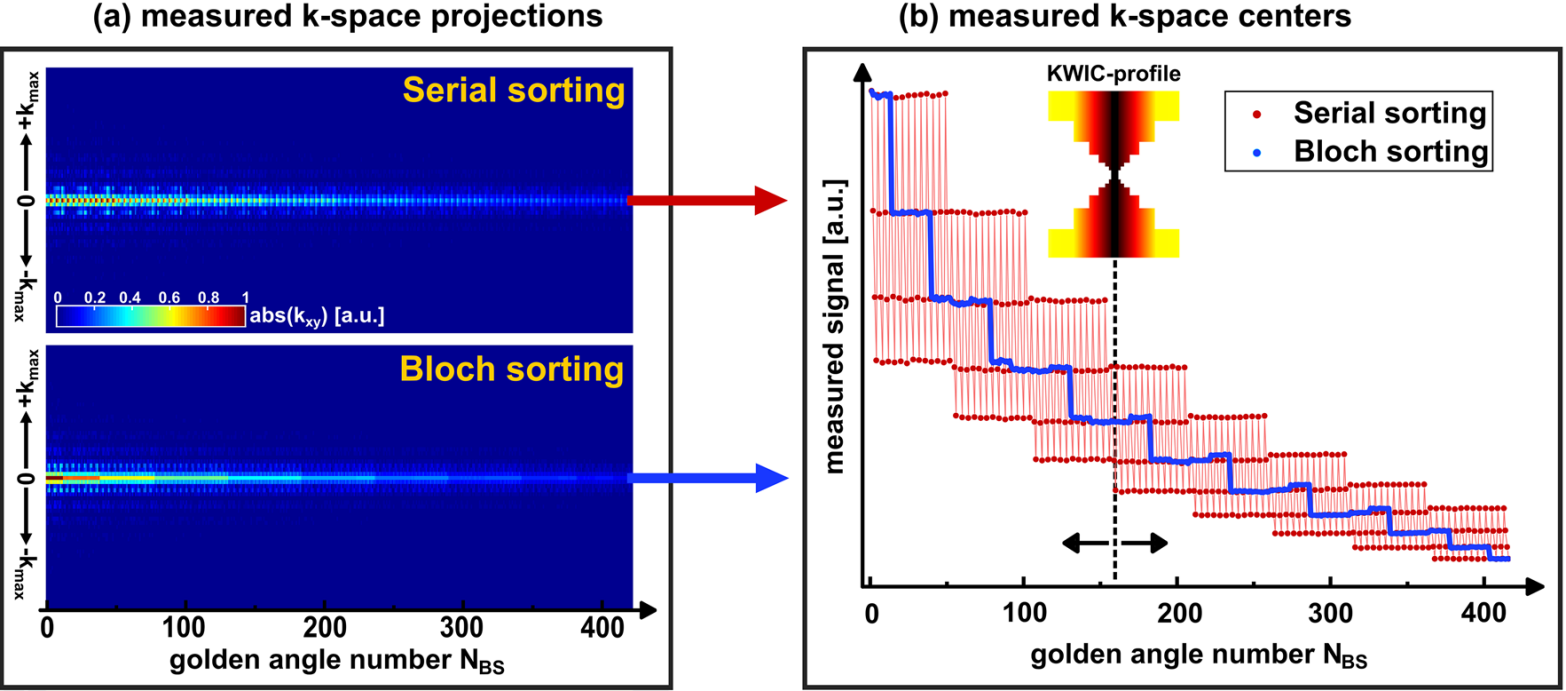
Comparison of the measured signal intensities for Serial sorting and Bloch sorting in the phantom experiment. **a** The k-space projections (absolute values) are visualized as heatmaps in order of the corresponding projection angles (golden angle number ${N}_{\upphi }$). **b** The signal intensities of the measured k-space centers are plotted versus the golden angle number ${N}_{\upphi }$. In the case of Bloch sorting the change of contrast for subsequent angles was minimized. The mean deviation from ideal sorting (monotonically decreasing) was 2%. For Serial sorting fast signal changes were observed

The results in Fig. 4 show the measured signal intensities over the projection angle of the respective readouts. Here, a good agreement with the predictions of the Bloch simulation (Fig. 3) can be observed. In the case of Bloch sorting, the intensities were sorted almost perfectly in a descending manner (98%). Only a few readouts were acquired in incorrect order. In the case of Serial sorting fast signal changes were observed due to the high flip angles used.

**Fig. 5:**
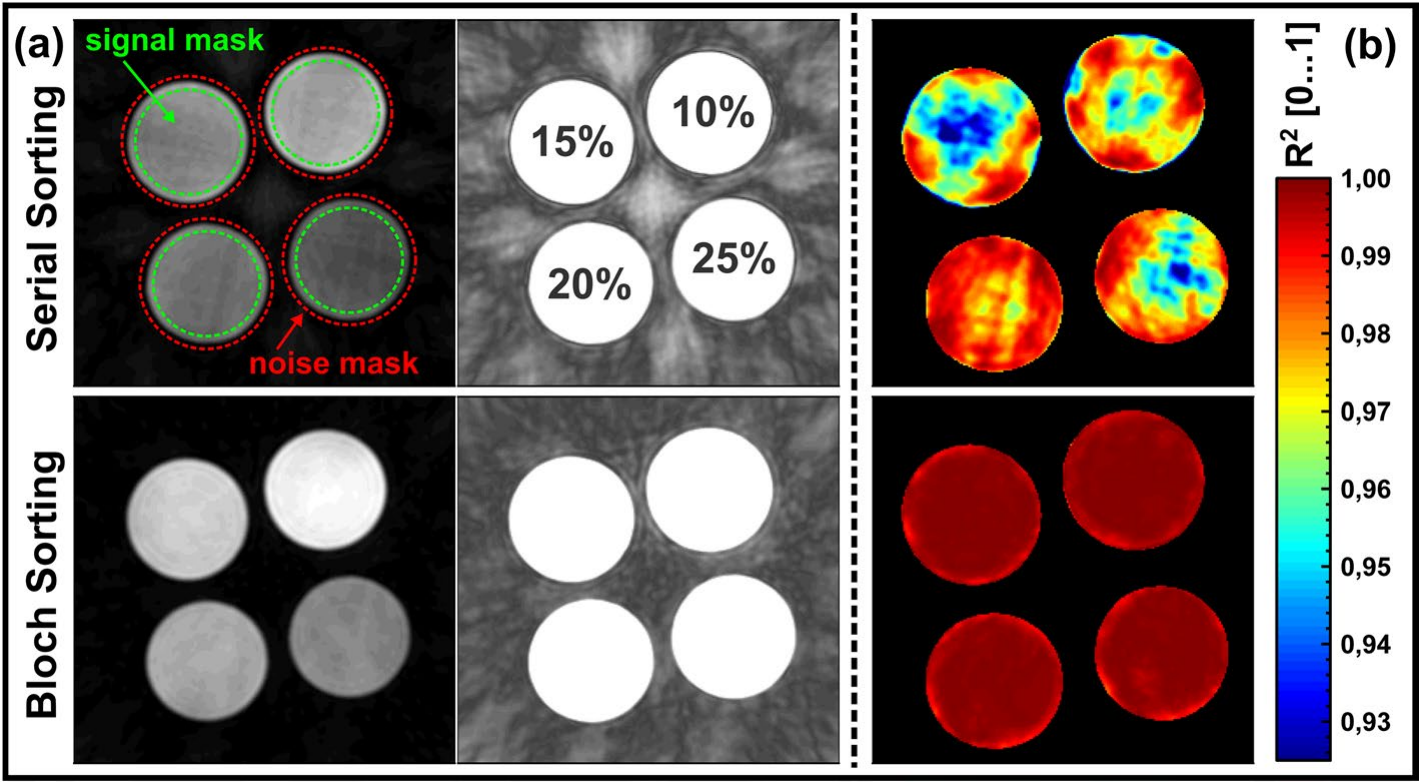
Artifact and SNR analysis of the novel Bloch simulation-based sampling scheme and the standard Serial sorting scheme. **a** Comparison of exemplary *T*_1ρ_-weighted images of the four BSA phantoms. Left column: the images are “normally” scaled (all images were acquired with an identical *t*_SL_ and *f*_SL_ combination). Right column: the scaling of the images is adapted to highlight artifacts. It is apparent, that Bloch sorting produces much less artifacts than the Serial sorting technique. The SNR values have been determined to SNR_Serial_ = 16.7 and SNR_Bloch_ = 44.6 for the images shown. **b** Comparison of *R*^2^ maps. The *R*^2^ values of the Bloch sorting method almost perfectly fit the value 1, while the other method only reaches lower values (${R}_{\mathrm{Serial}}^{2}=0.973$ and ${R}_{\mathrm{Bloch}}^{2}=0.999$). Structural shapes within the *R*^2^ maps are an indication of artifacts within the reconstructed *T*_1ρ_ weighted images and a measure of reduced image quality

Figure 5a shows the results of the artifact/SNR analysis. The images reveal clearly less artifacts using the optimized Bloch sampling scheme compared to the standard Serial sorting method, which is a result of the reduced contrast differences between acquired subsequent golden angles. The SNR values averaged over all reconstructed *T*_1ρ_ weighted images were SNR_Serial_ = 17.9 and SNR_Bloch_ = 39.3. This represents a SNR increase of 120% using the Bloch sorting scheme. The *R*^2^ map (coefficient of determination, Fig. 5b) also shows a better agreement with the fitted exponential *T*_1ρ_ decay, since the *R*^2^ values almost perfectly fit the value 1 using Bloch sorting. In the *R*^2^ map of the Serial sorting scheme, certain structural shapes are visible, which might be caused by the artifacts within the reconstructed *T*_1ρ_ weighted images. Hence, the *R*^2^ maps also represents a measure of potential artifacts. The mean *R*^2^ values were determined to be ${R}_{\mathrm{Serial}}^{2}=0.973$ and ${R}_{\mathrm{Bloch}}^{2}=0.999$.

**Fig. 6:**
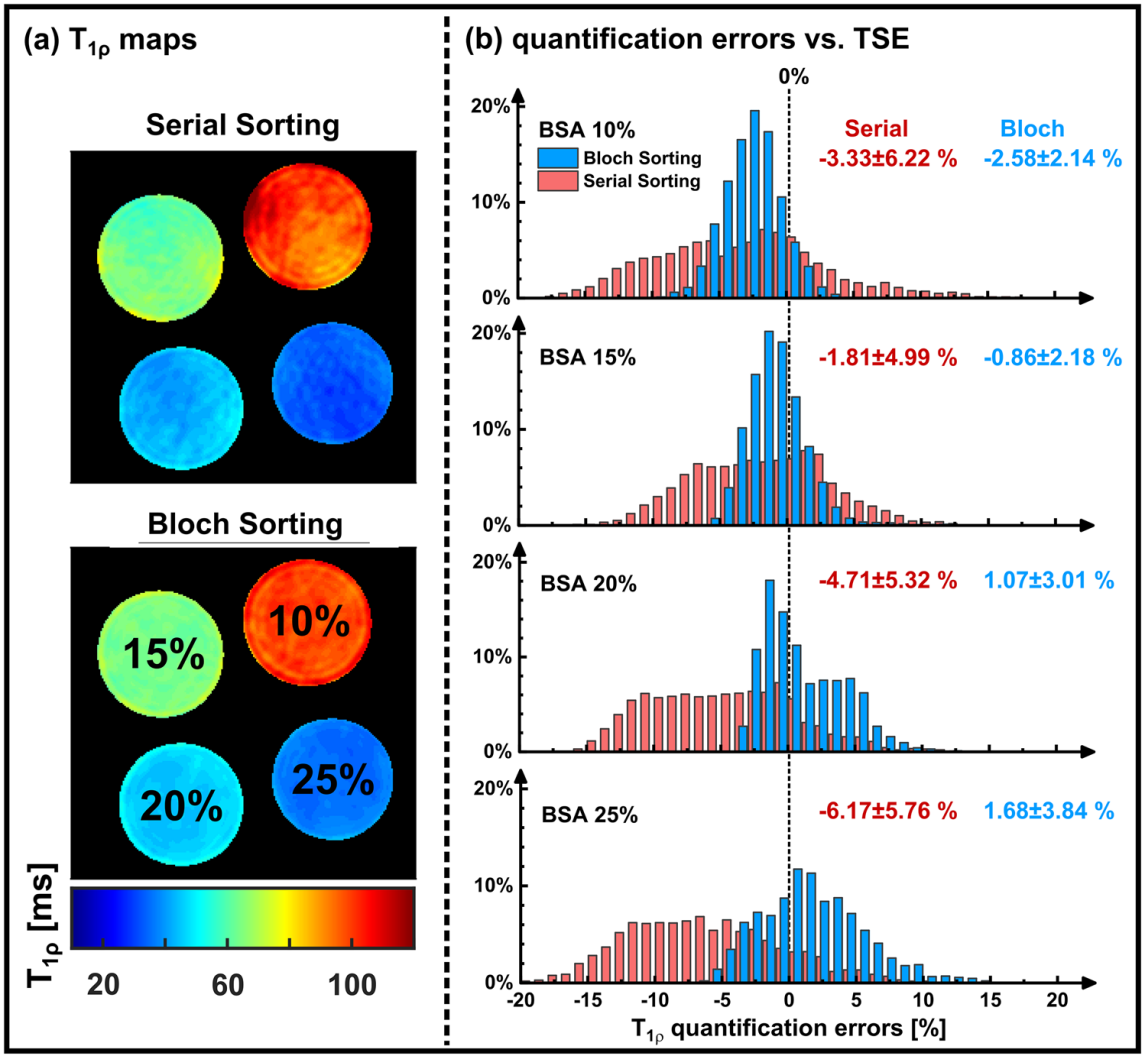
*T*_1ρ_ quantification of Serial Sorting and Bloch Sorting. **a** Calculated *T*_1ρ_ maps of the four BSA phantoms. Streaking artifacts were clearly reduced with Bloch Sorting. **b** Comparison of quantification errors using the TSE reference method. For the individual BSA tubes, the relative error in relation to the TSE reference was pixel-wise evaluated. The accuracy and precision has been improved by Bloch sorting for all phantoms. However, a slight underestimation (low BSA concentrations) and overestimation (high BSA concentrations) was obtained

The resulting T_1ρ_ maps for both sampling schemes are shown in Fig. 6a. An improved image quality for Bloch sorting is also visible here, since fewer streaking artifacts can be seen in the phantoms. The results of the *T*_1ρ_ quantification accuracy and precision measurements are illustrated in Fig. 6b. The quantification errors compared to the TSE reference have been determined for the individual BSA tubes using a pixel-wise evaluation (Table 1). The relative error averaged over all phantoms was − 4.01 ± 5.57% (mean value ± standard deviation) for Serial sorting and − 0.17 ± 2.79% for Bloch sorting. The optimized Bloch sorting scheme achieves a considerable improvement in accuracy (+ 56%) and precision (+ 49%). However, for the individual tubes, Bloch sorting showed a tendency to slightly underestimate long *T*_1ρ_ times and overestimate short times (Table 1).

**Table 1:** *T*_1ρ_ quantification of Serial sorting and Bloch sorting

BSA concentration	*R*^2^ Serial [0…1]	*R*^2^ Bloch [0…1]	Δ*Q* Serial [%]	ΔQ Bloch [%]	Improvement [%]	
					Accuracy	Precision
10%	0.974 ± 0.013	0.999 ± 0.001	− 3.33 ± 6.22	− 2.58 ± 2.14	+ 23	+ 66
15%	0.962 ± 0.018	0.999 ± 0.001	− 1.81 ± 4.99	− 0.86 ± 2.18	+ 52	+ 56
20%	0.986 ± 0.008	0.999 ± 0.001	− 4.71 ± 5.32	1.07 ± 3.01	+ 77	+ 43
25%	0.969 ± 0.015	0.999 ± 0.001	− 6.17 ± 5.76	1.68 ± 3.84	+ 73	+ 33
Mean	0.973 ± 0.016	0.999 ± 0.001	− 4.01 ± 5.57	− 0.17 ± 2.79	+ 56	+ 49

Comparison of Serial sorting and Bloch sorting in the phantom experiments. The *R*^2^ values and the relative quantification errors Δ*Q* for the individual BSA phantoms were examined. The results of the TSE sequence were used as a reference. The improvement in accuracy and precision achieved by Bloch sorting (vs Serial sorting) was determined by the respective quantification errors

**Fig. 7:**
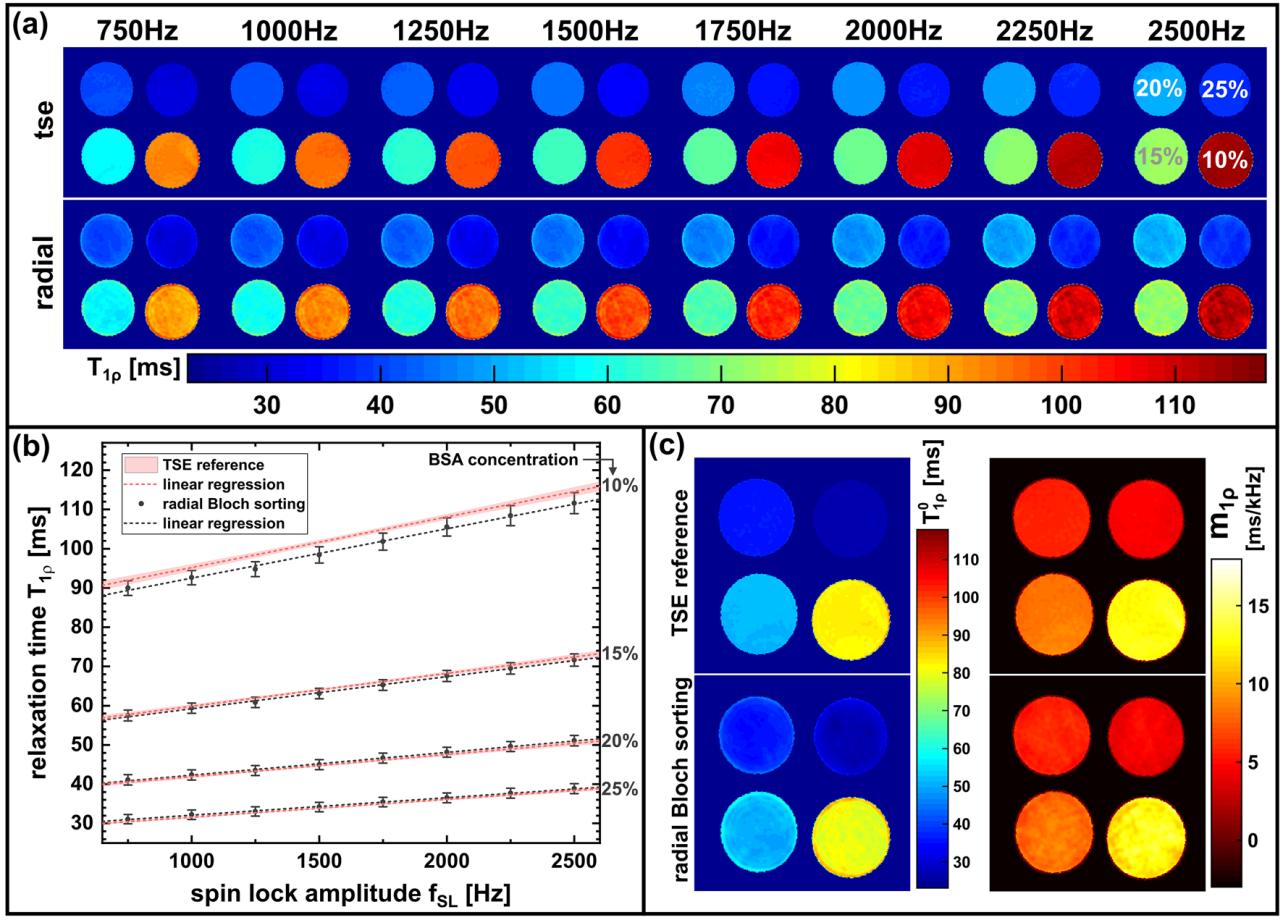
Comparison of the radial Bloch sorting method with the Cartesian TSE reference gold standard. **a** Results of *T*_1ρ_ mapping using different SL amplitudes (*f*_SL_ = 750…2500 Hz). Both methods clearly show *T*_1ρ_ dispersion for all BSA probes. **b** Mean *T*_1ρ_ values and standard deviation of the different sample probes plotted versus the SL amplitude. The calculated *T*_1ρ_ values of our new method are in good agreement with the reference TSE values (shaded area, mean ± std). **c** Heat maps of the dispersion offset ${\mathrm{T}}_{1\uprho }^{0}$ and the dispersion slope ${\mathrm{m}}_{1\uprho }$ calculated using a linear dispersion model (Eq. 3). The results of the radial Bloch sorting scheme and the TSE reference measurements are in good agreement and show a comparable image quality. Only at the edges of the sample tubes, some deviations are visible using the Bloch sorting scheme. This might be caused by data undersampling and the KWIC-filtered view-sharing technique

The results of the *T*_1ρ_ dispersion investigations are shown in Fig. 7a. The mean T_1ρ_ values of the four sample probes are plotted versus the SL amplitude for our radial Bloch sorting scheme and the reference values obtained from the TSE measurement (Fig. 7b). The quantification error was averaged for all SL amplitudes and determined to be − 0.46 ± 1.84%. In Fig. 7c the calculated dispersion slope maps and dispersion offset maps using the linear dispersion model (Eq. 3) are shown. The obtained dispersion slopes ${m}_{1\rho }$ and offsets ${T}_{1\uprho }^{0}$ for the different sample probes and the comparison with the reference TSE values are listed in Table 2. The dispersion behavior of the four samples can be clearly differentiated using our new radial sequence. For the dispersion slope a mean deviation of − 1.3% and a maximum deviation of − 2.8% has been observed. Mean deviation of the dispersion offset was − 0.2% and the maximum deviation was − 3.4%. For the dispersion analysis, good agreement with a linear model could be determined in the range of the SL amplitudes used. The coefficient of determination of the linear regression averaged at least 0.993.

**Table 2:** Dispersion analysis of the phantom experiments

BSA concentration	TSE reference	Radial Bloch sorting
Dispersion slope *m*_1ρ_ [ms/kHz]		
10%	12.81 ± 0.35	12.67 ± 0.84 (− 1.1%)
15%	8.42 ± 0.26	8.20 ± 0.50 (− 2.7%)
20%	5.73 ± 0.13	5.80 ± 0.33 (+ 1.3%)
25%	4.59 ± 0.16	4.46 ± 0.37 (− 2.8%)
Dispersion offset *T*_1ρ_^0^ [ms]		
10%	82.60 ± 0.63	79.81 ± 1.61 (− 3.4%)
15%	51.51 ± 0.39	51.03 ± 1.41 (− 0.9%)
20%	36.11 ± 0.20	36.50 ± 1.38 (+ 1.1%)
25%	27.00 ± 0.20	27.63 ± 1.26 (+ 2.3%)
Coefficient of determination *R*^2^ [0…1]		
10%	0.989 ± 0.004	0.994 ± 0.003
15%	0.993 ± 0.003	0.996 ± 0.002
20%	0.991 ± 0.004	0.996 ± 0.002
25%	0.991 ± 0.004	0.996 ± 0.002

Calculated dispersion slopes *m*_1ρ_ and dispersion offsets ${T}_{1\uprho }^{0}$ of the four sample probes for the TSE reference measurement and the radial sequence using Bloch sorting. For both methods, the dispersion values decrease with increasing BSA concentration. The values in brackets represent the percentage deviation from the TSE reference with a maximum deviation of − 2.8% for the dispersion slope and − 3.4% for the dispersion offset. The high *R*^2^ values of consistently > 0.98 indicate a very good agreement of the acquired data with the linear dispersion model

### In vivo measurements

**Fig. 8:**
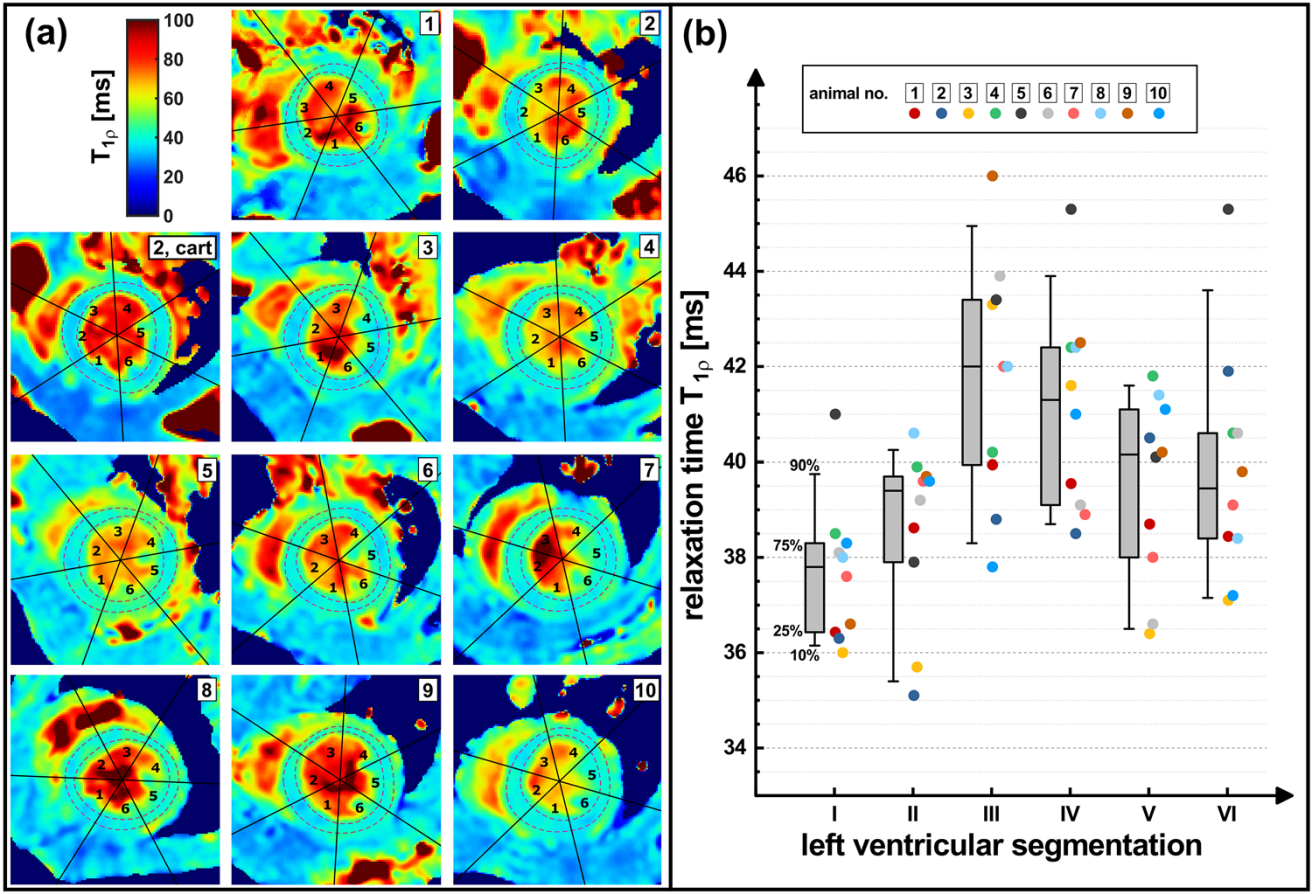
Results of in vivo *T*_1ρ_ mapping in *N* = 10 mice. **a** Images of the acquired *T*_1ρ_ maps (short-axis view, isotropic resolution 250 μm) with identical SL amplitudes of 1500 Hz. All maps were segmented in the left ventricle according to the AHA model. In animal 2, a map was also calculated from a fully sampled Cartesian data set. **b** The results of the segmentation are shown in a box plot. In the segments 3 and 4 the values are slightly increased. Segment 1 tends to have the lowest values

The calculated *T*_1ρ_ maps of the *N* = 10 mice are shown in Fig. 8. A segmentation of the left ventricle according to the AHA model was carried out for each map. The results are listed in Table 3 for all animals (physiological parameters in the supplementary material, Online Table 2). Here, the breathing cycle was 1460 ± 154 ms and the cardiac cycle length was 137.3 ± 5.9 ms averaged over all animals. The global left ventricular *T*_1ρ_ was 39.5 ± 1.2 ms for *f*_SL_ = 1500 Hz. Slightly increased *T*_1ρ_ values could be found in the segments 3 (41.7 ± 2.5 ms) and 4 (41.1 ± 2.1 ms). The comparison of the Cartesian reference measurement (37.8 ± 3.7 ms) with the accelerated radial method (38.3 ± 3.3 ms) revealed a small deviation of + 1.3%.

**Table 3:** Results of in vivo *T*_1ρ_ mapping in mice

	Relaxation time *T*_1ρ_ [ms]						
Animal	LV	AHA 1	AHA 2	AHA 3	AHA 4	AHA 5	AHA 6
1	38.5 ± 0.5	36.4 ± 0.7	38.6 ± 0.4	39.9 ± 1.7	39.6 ± 1.3	38.7 ± 0.6	38.4 ± 0.6
2	38.3 ± 3.3	36.3 ± 1.6	35.1 ± 1.5	38.8 ± 2.8	38.5 ± 2.0	40.5 ± 2.5	41.9 ± 3.5
3	37.9 ± 3.7	36.0 ± 2.0	35.7 ± 1.8	43.3 ± 2.3	41.6 ± 3.4	36.4 ± 2.9	37.1 ± 2.3
4	40.5 ± 2.6	38.5 ± 1.8	39.9 ± 1.9	40.2 ± 2.7	42.4 ± 2.1	41.8 ± 2.4	40.6 ± 2.6
5	41.8 ± 4.0	41.0 ± 2.2	37.9 ± 1.9	43.4 ± 3.2	45.3 ± 5.2	40.1 ± 2.1	45.3 ± 1.8
6	39.4 ± 3.2	38.1 ± 1.9	39.2 ± 1.7	43.9 ± 2.2	39.1 ± 2.9	36.6 ± 2.2	40.6 ± 2.6
7	39.2 ± 2.5	37.6 ± 2.4	39.6 ± 1.9	42.0 ± 1.5	38.9 ± 2.4	38.0 ± 1.1	39.1 ± 2.4
8	40.4 ± 3.3	38.0 ± 2.5	40.6 ± 3.4	42.0 ± 3.6	42.4 ± 2.6	41.4 ± 1.7	38.4 ± 2.7
9	40.0 ± 3.6	36.6 ± 3.0	39.7 ± 2.4	46.0 ± 2.7	42.5 ± 2.5	40.2 ± 2.6	39.8 ± 1.7
10	39.1 ± 2.5	38.3 ± 1.4	39.6 ± 1.7	37.8 ± 2.5	41.0 ± 2.5	41.1 ± 2.4	37.2 ± 2.4
Mean	39.5 ± 1.2	37.7 ± 1.5	38.6 ± 1.8	41.7 ± 2.5	41.1 ± 2.1	39.4 ± 1.9	39.7 ± 2.4

*T*_1ρ_ quantification results in *N* = 10 different animals for *f*_SL_ = 1500 Hz. The table shows the results in the individual AHA segments for *T*_1ρ_, as well as the mean values in the global left ventricular ROI (LV). In the supplementary material (Online Table 2) the results of the reproduction study, the comparison with the fully sampled Cartesian reference measurement and the monitored physiological parameters (cardiac cycle and breath cycle length) are listed

**Fig. 9:**
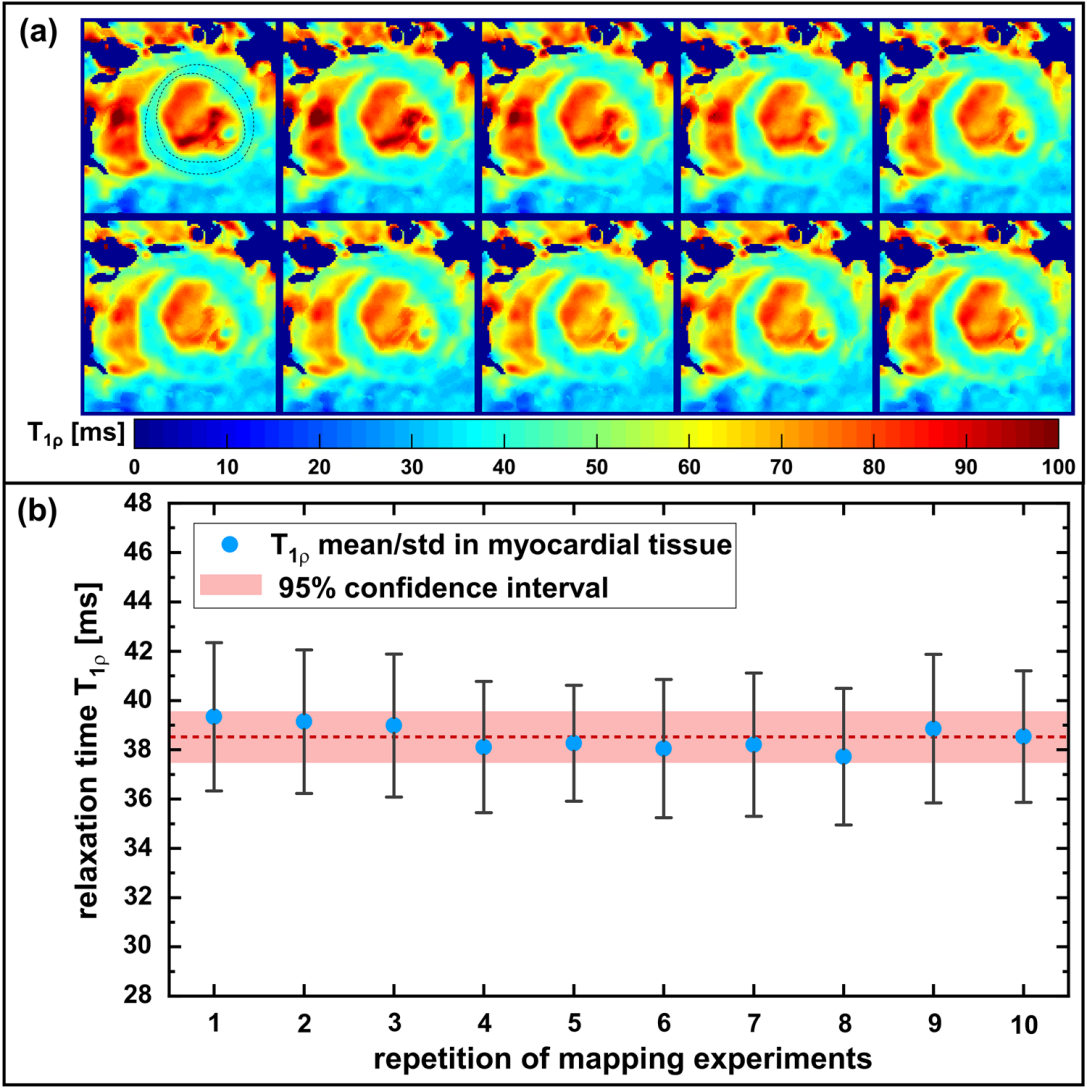
Results of the in vivo reproducibility measurements. **a** Images of the ten repeatedly acquired *T*_1ρ_ maps (short-axis view, isotropic resolution 250 μm) with identical SL amplitudes of 1500 Hz. All *T*_1ρ_ maps show the same image quality without any motion, flow or reconstruction artifacts. The dashed lines represent the chosen ROI for data analysis. **b** Mean (blue dots) and standard deviation (black lines) of the myocardial *T*_1ρ_ values of the ten repetitive measurements. Mean *T*_1ρ_ has been determined to 38.52 ± 0.54 ms (dotted red line and light red area)

In Fig. 9 the results of the in vivo reproducibility study are depicted. Figure 9a shows the ten repetitive *T*_1ρ_ maps, indicating a very good image quality. There are hardly any visual differences in the images. All structures are at the same position and the quantified *T*_1ρ_ values appear to be almost identical. A ROI-based analysis of the left ventricular myocardium shows a mean *T*_1ρ_ of 38.52 ms and only a small variation in the individual T_1ρ_ values with a standard deviation of ± 0.54 ms (Fig. 9b). The maximum deviation observed in the separate experiments was 2.1%.

**Fig. 10:**
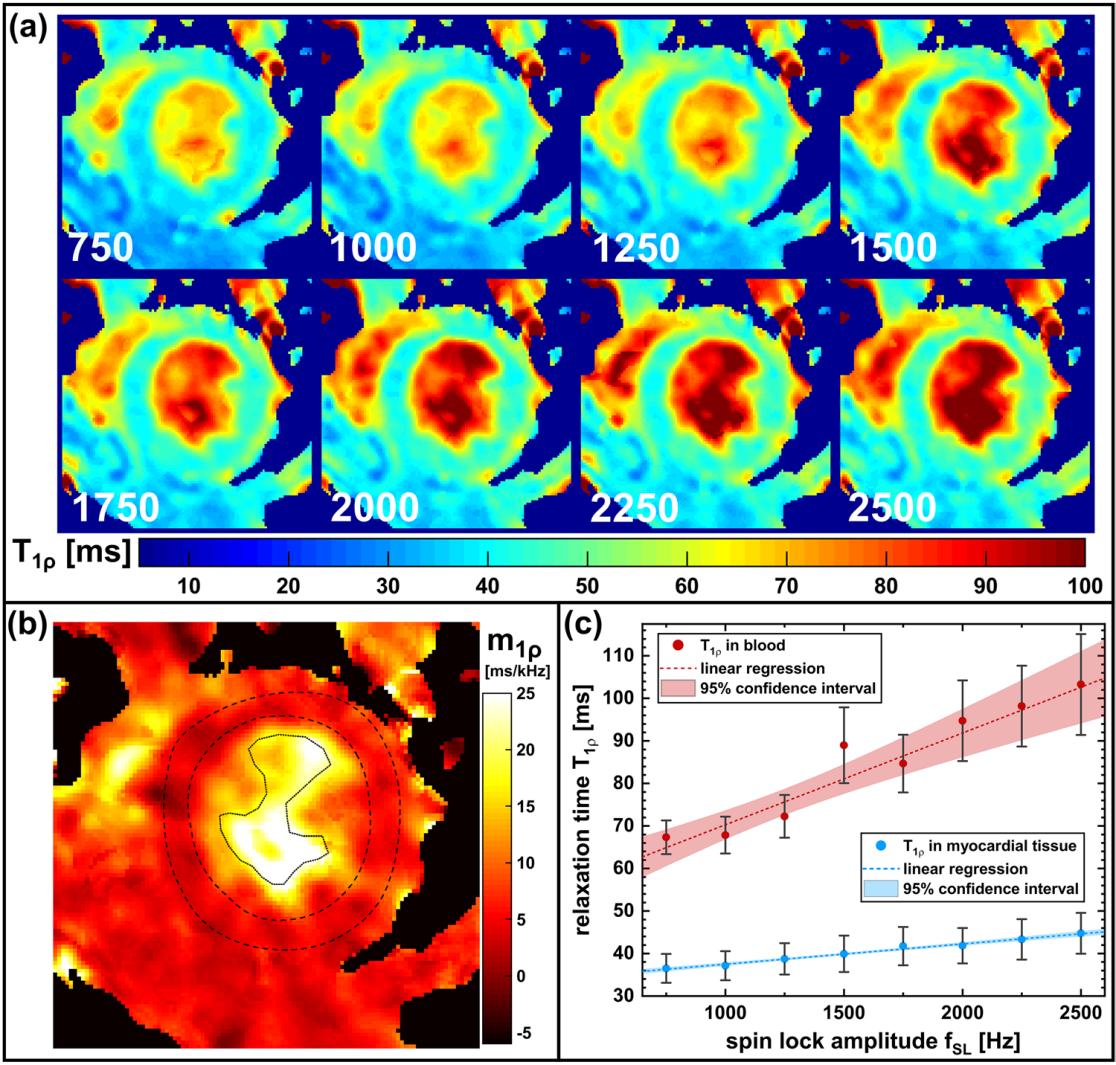
In vivo *T*_1ρ_ mapping including *T*_1ρ_ dispersion quantification (short-axis view, isotropic resolution 250 μm). **a** Exemplary *T*_1ρ_ maps acquired with different SL amplitudes (*f*_SL_ = 750 Hz…2500 Hz). The increase of *T*_1ρ_ with increasing *f*_SL_ is clearly visible. All *T*_1ρ_ maps show diagnostic image quality without any motion, flow or reconstruction artifacts. **b** Myocardial dispersion slope map. The image quality is also good with a slight blurring at the myocardial borders. The dashed lines represent the location of the chosen ROIs for the *T*_1ρ_ dispersion quantification of myocardium and the blood pool. **c** Analysis of the dispersion behavior of myocardial tissue and blood. The *T*_1ρ_ dispersion slopes are 4.76 ± 0.23 ms/kHz for left ventricular myocardium and 21.57 ± 2.56 ms/kHz for the left ventricular blood pool

Figure 10 shows the results of the in vivo *T*_1ρ_ dispersion quantification measurements. The *T*_1ρ_ maps show a very good image quality with minimal motion, flow or reconstruction artifacts. The *T*_1ρ_ dispersion map reveals a diagnostic image quality with little blurring at the myocardial borders. This might be caused by slightly different heart phases within the underlying *T*_1ρ_ maps. The *T*_1ρ_ dispersion slope has been determined to 4.76 ± 0.23 ms/kHz for left ventricular myocardium and 21.57 ± 2.56 ms/kHz for the left ventricular blood pool. The dispersion offset could be determined to be 32.73 ± 0.36 and 48.72 ± 3.41 ms, respectively.

## Discussion

In the current study, we introduced a fast cardiac *T*_1ρ_ quantification technique that enables high-resolution in vivo *T*_1ρ_ mapping and even *T*_1ρ_ dispersion mapping in small animals. The method consisted of three concepts; Signal maximization using high flip angles, a specially adapted radial data sampling (which was optimized using Bloch simulations) and a KWIC-filtered view-sharing technique for efficient image reconstruction. With these concepts, an appropriate data undersampling (factor 32:13 ≈ 2.5, 13 preparations for radial sampling, 32 = 128/4 preparations for Cartesian sampling) is possible requiring only a fraction of the commonly needed data for the acquisition of *T*_1ρ_ weighted images.

The optimized radial sequence ensures a high signal-to-noise-ratio and is combined with a very efficient sampling strategy that accelerates data acquisition while reducing the occurrence of artifacts and preventing incorrect *T*_1ρ_ weighting. The main reason for this is that the pre-calculation of the expected signal intensity using Bloch simulations avoids strong changes in the signal levels of consecutive spokes. This results in an improved image quality with minor artifacts and an SNR increase of 120% in contrast to a conventional Serial sorting scheme of golden angles. Besides, the optimized Bloch sampling strategy reveals a higher accuracy and precision of the calculated *T*_1ρ_ values compared to Serial sorting. The *T*_1ρ_ quantification accuracy of Bloch sorting has proven in phantom measurements to be very high with a mean deviation of − 0.46 ± 1.84% compared to a TSE reference measurement. We observed the largest quantification errors in the phantom with 10% BSA concentration. Compared to the other phantoms, this has a much longer *T*_1ρ_ relaxation time. Here the influence of the KWIC filter could possibly affect the contrasts. As a result, systematically shorter relaxation times were measured (≈ − 3%). However, this shift did not affect the quantification of the dispersion slope and offset and the *T*_1ρ_ range of this phantom is hardly relevant for the characterization of myocardial tissue.

As described by Song et al. [31], the variation of signal intensities between the radial profiles possibly results in different relative proportions of high and low spatial frequencies for different weighted images. This effect depends on the design of the KWIC filter and was examined in the supplementary material. If the Nyquist criterion is not met in all regions of k-space (Nyquist factor *f*_nyq_ < 1), undersampling artifacts and reduced SNR occur (Online Fig. 2). Opposed, excessive sharing of different contrasts (*f*_nyq_ > 2) results in edge blurring and edge sharpening (Online Fig. 3). As the results in the supplementary material show, the T_1ρ_ quantification accuracy is also affected by the choice of the KWIC filter design (Online Figs.4 and 5). However, in the range chosen in this work (*f*_nyq_ = 1.1) the accuracy is stable and only shows systematic errors for the phantom with the highest relaxation time. Using moderate Nyquist factors *f*_nyq_ = 1.0…1.5, the influence of the KWIC filter design on the quantification is small (± 0.3%) and can be neglected in the context of in vivo experiments.

In this work constant high flip angles (40°) were used for the gradient echo readout. For small flip angles, Bloch sorting can be avoided. However, in a previous work we could show that the resulting image quality is significantly reduced using an echo number based sorting and 10° flip angles, for example [36]. Yet, it is also possible to use ramped flip angles after the preparation. This could prevent the formation of signal plateaus and, if the RF pulse accuracy is well calibrated, the Bloch sorting could be omitted. However, different *T*_1ρ_ weightings require different ramps, whereby the first readout must always be carried out using the same flip angle in order not to impair *T*_1ρ_ quantification.

In the in vivo experiments, a good comparability of the *T*_1ρ_ values in the left ventricle could be achieved for different animals. The *T*_1ρ_ values determined at *f*_SL_ = 1500 Hz varied between 37.9 and 41.8 ms. Musthafa et al. obtained values in the range 32…38 ms for *f*_SL_ = 1250 Hz and *B*_0_ = 9.4 T [20]. The deviation could be explained by the *T*_1ρ_ dispersion, since, according to basic relaxation theory, smaller values are expected for decreasing *f*_SL_. In addition, a high goodness of the mono-exponential *T*_1ρ_ fit could be observed in most of the measurements. The values in the left ventricle were *R*^2^ = 0.993 on average. The slightly increased *T*_1ρ_ values in the segments 3 (+ 5.6%) and 4 (+ 4.1%) may have been caused by partial volume effects, since the wall thickness was also reduced here. However, streaking and blurring artifacts could also have an impact. The fully sampled Cartesian measurement shows a slightly improved image quality compared to the accelerated radial technique. However, the quantification in the respective segments is in good agreement (+ 1.9 ± 3.3%) and the deviation in the global left ventricular ROI was only + 1.3%. It also needs to be considered that the cardiac phases were not exactly identical. The cycle length for the radial acquisition was 131.5 ± 1.2 ms and for the Cartesian measurement 153.2 ± 0.7 ms. This can also be seen in the contrast of the blood pool.

The in vivo reproducibility study showed, that our method enables a high degree of comparability for successive measurements, which is also crucial for dispersion imaging. This is a consequence of the radial readout that is more robust against moving tissues and blood flow. However, the main reason might be the very short measurement time of around 2.5 min. Alternative methods that require longer measurement times can lead to problems in the stability of the animal under investigation due to variations in the cardiac and respiratory cycle. This would require additional compensation and/or correction mechanisms, which is quite complex and hampers the practicality of cardiac *T*_1ρ_ and/or *T*_1ρ_ dispersion mapping.

Furthermore, a new approach for quantifying the dispersion behavior was introduced in this work. Due to the high acceleration of the radial data acquisition, the measurement could be accomplished in 20 min. In this total measurement time, only relatively small variations in the physiological parameters were observed. The mean cardiac cycle length was 141.5 ± 3.9 ms while the breathing cycle was 1430 ± 63 ms. In comparison, a fully sampled Cartesian method would have taken 50 min, potentially leading to higher variations. The determination of the dispersion slope m_1ρ_ and the offset ${T}_{1\uprho }^{0}$ was based on the approach of Yin et al. [19], in which the difference ${T}_{1\uprho }^{0}-{T}_{1\uprho }({f}_{\mathrm{SL}})$ is presented as a potential myocardial fibrosis index. The calculation of a slope has an advantage over a differential measurement because the value is thus normalized. In addition, the determination of the dispersion slope based on eight independent *T*_1ρ_ maps increases the accuracy compared to the previously described method of Yin et al. [19]. Nevertheless, it is necessary to ensure that the linear range of *T*_1ρ_ dispersion is not exceeded, since it is known from relaxation theory that *T*_1ρ_ dispersion is formally more complex than a linear model [10, 11]. In [19] the range *f*_SL_ = 0…510 Hz was used for dispersion quantification, although it was not explained why this range is particularly suitable. Future studies must therefore primarily investigate which ranges of SL amplitudes are most suitable for calculating a fibrosis index.

For the translation of the method to human studies, the SAR (specific absorption rate) is a limiting factor. In this study, relatively high SL amplitudes were used, which show a low susceptibility to *B*_0_ inhomogeneities [27, 28]. These amplitudes are hardly feasible using radiofrequency amplifiers available on clinical scanners, which ultimately limits routine clinical applications [29]. The concept of Bloch sorting can, however, be transferred, whereby far more readouts would be possible after the preparation due to the lower heart rate in humans. Here the optimal flip angle arises with significantly lower values, which could possibly be utilized for SAR reduction.

A drawback of our study is that we are not yet able to present results in fibrotic myocardial tissue. The quantification of the *T*_1ρ_ dispersion is of particular interest for future studies. Measurements in the infarct model, in which the dispersion is to be examined in detail, are planned. Besides, work is in progress to develop an extension of the sequence design presented in this study. Here, not only one *T*_1ρ_ map should be acquired in a single scan, but the whole data set required for dispersion mapping, since separate maps can ultimately lead to quantification errors due to misregistration and cardiac motion. For this purpose, an optimization of the radial sampling pattern is also considered based on the Bloch sorting principle.

## Conclusion

In conclusion, our new *T*_1ρ_ quantification technique represents a reasonable tool for cardiac *T*_1ρ_ mapping and *T*_1ρ_ dispersion imaging. The method is feasible for improved cardiac tissue characterization and possibly enables enhanced diagnostics for several diseases as it might identify diffuse fibrosis. Due to its very short measurement time, stability and robustness, this method might be included in a common in vivo measurement protocol for small animals without major difficulty.

## Supplementary Information

Below is the link to the electronic supplementary material.

Supplementary file1 (PDF 1530 KB)

## Data Availability

The datasets used and/or analyzed during the current study are available from the corresponding author on reasonable request.

## References

[1] Ferreira VM, Schulz-Menger J, Holmvang G, Kramer CM, Carbone I, Sechtem U, Kindermann I, Gutberlet M, Cooper LT, Liu P, Friedrich MG (2018). Cardiovascular magnetic resonance in nonischemic myocardial inflammation: expert recommendations. J Am Coll Cardiol.

[2] Lewis AJM, Burrage MK, Ferreira VM (2020). Cardiovascular magnetic resonance imaging for inflammatory heart diseases. Cardiovasc Diagn Ther.

[3] Everett RJ, Stirrat CG, Semple SI, Newby DE, Dweck MR, Mirsadraee S (2016). Assessment of myocardial fibrosis with T1 mapping MRI. Clin Radiol.

[4] Ferreira VM, Piechnik SK (2020). CMR parametric mapping as a tool for myocardial tissue characterization. Korean Circ J.

[5] Gensler D, Mörchel P, Fidler F, Ritter O, Quick HH, Ladd ME, Bauer WR, Ertl G, Jakob PM, Nordbeck P (2015). Myocardial T1: quantification by using an ECG-triggered radial single-shot inversion-recovery MR imaging sequence. Radiology.

[6] Piechnik SK, Neubauer S, Ferreira VM (2018). State-of-the-art review: stress T1 mapping-technical considerations, pitfalls and emerging clinical applications. Magn Reson Mater Phys.

[7] Haaf P, Garg P, Messroghli DR, Broadbent DA, Greenwood JP, Plein S (2016). Cardiac T1 Mapping and Extracellular Volume (ECV) in clinical practice: a comprehensive review. J Cardiovasc Magn Reson.

[8] Cameron D, Vassiliou VS, Higgins DM, Gatehouse PD (2018). Towards accurate and precise T1 and extracellular volume mapping in the myocardium: a guide to current pitfalls and their solutions. Magn Reson Mater Phy.

[9] Redfield AG (1955). Nuclear magnetic resonance saturation and rotary saturation in solids. Phys Rev.

[10] Bull TE (1992). Relaxation in the rotating frame in liquids. Prog Nucl Magn Reson Spectrosc.

[11] Gilani IA, Sepponen R (2016). Quantitative rotating frame relaxometry methods in MRI. NMR Biomed.

[12] Duvvuri U, Goldberg AD, Kranz JK, Hoang L, Reddy R, Wehrli FW, Wand AJ, Englander SW, Leigh JS (2001). Water magnetic relaxation dispersion in biological systems: the contribution of proton exchange and implications for the noninvasive detection of cartilage degradation. Proc Natl Acad Sci USA.

[13] Mäkelä HI, Gröhn OH, Kettunen MI, Kauppinen RA (2001). Proton exchange as a relaxation mechanism for T1 in the rotating frame in native and immobilized protein solutions. Biochem Biophys Res Commun.

[14] Adela SV, Regatte RR, Wheaton AJ, Borthakur A, Reddy R (2004). Reduction of residual dipolar interaction in cartilage by spin lock technique. Magn Reson Med.

[15] Witschey WR, Pilla JJ, Ferrari G, Koomalsingh K, Haris M, Hinmon R, Zsido G, Gorman JH, Gorman RC, Reddy R (2010). Rotating frame spin lattice relaxation in a swine model of chronic, left ventricular myocardial infarction. Magn Reson Med.

[16] Muthupillai R, Flamm SD, Wilson JM, Pettigrew RI, Dixon WT (2004). Acute myocardial infarction: tissue characterization with T1rho-weighted MR imaging–initial experience. Radiology.

[17] Witschey WR, Zsido GA, Koomalsingh K, Kondo N, Minakawa M, Shuto T, McGarvey JR, Levack MM, Contijoch F, Pilla JJ, Gorman JH, Gorman RC (2012). In vivo chronic myocardial infarction characterization by spin locked cardiovascular magnetic resonance. J Cardiovasc Magn Reson.

[18] Han Y, Liimatainen T, Gorman RC, Witschey WR (2014). Assessing myocardial disease using T1ρ MRI. Curr Cardiovasc Imaging Rep.

[19] Yin Q, Abendschein D, Muccigrosso D, O'Connor R, Goldstein T, Chen R, Zheng J (2017). A non-contrast CMR index for assessing myocardial fibrosis. Magn Reson Imaging.

[20] Musthafa HS, Dragneva G, Lottonen L, Merentie M, Petrov L, Heikura T, Ylä-Herttuala E, Ylä-Herttuala S, Gröhn O, Liimatainen T (2013). Longitudinal rotating frame relaxation time measurements in infarcted mouse myocardium in vivo. Magn Reson Med.

[21] Stoffers RH, Madden M, Shahid M, Contijoch F, Solomon J, Pilla JJ, Gorman JH, Gorman RC, Witschey WRT (2017). Assessment of myocardial injury after reperfused infarction by T1ρ cardiovascular magnetic resonance. J Cardiovasc Magn Reson.

[22] Yla-Herttuala E, Laidinen S, Laakso H, Liimatainen T (2018). Quantification of myocardial infarct area based on TRAFFn relaxation time maps—comparison with cardiovascular magnetic resonance late gadolinium enhancement, T1ρ and T2 in vivo. J Cardiovasc Magn Reson.

[23] Nordbeck P, Hiller KH, Fidler F, Warmuth M, Burkard N, Nahrendorf M, Jakob PM, Quick HH, Ertl G, Bauer WR, Ritter O (2011). Feasibility of contrast-enhanced and nonenhanced MRI for intraprocedural and postprocedural lesion visualization in interventional electrophysiology: animal studies and early delineation of isthmus ablation lesions in patients with typical atrial flutter. Circ Cardiovasc Imaging.

[24] Herrmann S, Fries B, Salinger T, Liu D, Hu K, Gensler D, Strotmann J, Christa M, Beer M, Gattenlöhner S, Störk S, Voelker W, Bening C, Lorenz K, Leyh R, Frantz S, Ertl G, Weidemann F, Nordbeck P (2018). Myocardial fibrosis predicts 10-year survival in patients undergoing aortic valve replacement. Circ Cardiovasc Imaging.

[25] Müntze J, Salinger T, Gensler D, Wanner C, Nordbeck P (2018). Treatment of hypertrophic cardiomyopathy caused by cardiospecific variants of Fabry disease with chaperone therapy. Eur Heart J.

[26] Wáng YX, Zhang Q, Li X, Chen W, Ahuja A, Yuan J (2015). T1ρ magnetic resonance: basic physics principles and applications in knee and intervertebral disc imaging. Quant Imaging Med Surg.

[27] Chen W (2015). Errors in quantitative T1rho imaging and the correction methods. Quant Imaging Med Surg.

[28] Gram M, Seethaler M, Gensler D, Oberberger J, Jakob PM, Nordbeck P (2021). Balanced spin lock preparation for B1-insensitive and B0-insensitive quantification of the rotating frame relaxation time T1ρ. Magn Reson Med.

[29] Qi H, Bustin A, Kuestner T, Hajhosseiny R, Cruz G, Kunze K, Neji R, Botnar RM, Prieto C (2020). Respiratory motion-compensated high-resolution 3D whole-heart T1ρ mapping. J Cardiovasc Magn Reson.

[30] Winkelmann S, Schaeffter T, Koehler T, Eggers H, Doessel O (2007). An optimal radial profile order based on the Golden Ratio for time-resolved MRI. IEEE Trans Med Imaging.

[31] Song HK, Dougherty L (2000). k-space weighted image contrast (KWIC) for contrast manipulation in projection reconstruction MRI. Magn Reson Med.

[32] Winter P, Kampf T, Holley X, Gutjahr FT, Meyer CB, Bauer WR, Jakob PM, Herold V (2016). Self-navigation under non-steady-state conditions: Cardiac and respiratory self-gating of inversion recovery snapshot FLASH acquisitions in mice. Magn Reson Med.

[33] Li X, Han ET, Busse RF, Majumdar S (2008). In vivo T(1rho) mapping in cartilage using 3D magnetization-prepared angle-modulated partitioned k-space spoiled gradient echo snapshots (3D MAPSS). Magn Reson Med.

[34] Fessler JA, Sutton BP (2003). Nonuniform fast Fourier transforms using min-max interpolation. IEEE Trans Signal Process.

[35] Fessler JA. Michigan Image Reconstruction Toolbox (MIRT). http://web.eecs.umich.edu/~fessler/irt/irt/. Accessed 10 Nov 2020

[36] Gram M, Gensler D, Winter P, Seethaler M, Nordbeck P, Jakob PM (2020) Fast T1rho mapping in mice using an optimized Bloch simulation based radial sampling pattern. Proc Soc Magn Reson Med. ISMRM Annual Meeting. Virtual Conference. #2054

