# Supplementary material for: Fast myocardial T1ρ mapping in mice using k-space weighted image contrast and a Bloch simulation-optimized radial sampling pattern
Source: MAGMA. 2021 Sep 7;35(2):325–40. doi: 10.1007/s10334-021-00951-y (PMC8995242; doi:10.1007/s10334-021-00951-y)
Supplement: Supplementary file 1 — Supplementary file1 (PDF 1530 KB) [file 10334_2021_951_MOESM1_ESM.pdf]

Supplementary material:

Fast myocardial $T_{1\rho}$ mapping in mice using k-space weighted image contrast and a Bloch simulation-optimized radial sampling pattern

M. Gram[1,2], D. Gensler[1,3], P. Winter[1,2], M. Seethaler[2,3], P.A. Arias-Loza[3,4], J. Oberberger[1,2], P.M. Jakob[2], P. Nordbeck[1,3]

[1]Department of Internal Medicine I, University Hospital Würzburg, Würzburg

[2]Experimental Physics 5, University of Würzburg, Würzburg

[3]Comprehensive Heart Failure Center (CHFC), University Hospital Würzburg, Würzburg

[4]Department of Nuclear Medicine, University Hospital Würzburg, Würzburg

Address for correspondence: Dr. Peter Nordbeck M.D., University Hospital Würzburg, Department of Internal Medicine I, Oberdürrbacher Str. 6, Würzburg, DE D-97080, nordbeck_p@ukw.de, https://orcid.org/0000-0002-2560-4068

This document contains the following supplementary sections and files:

Sections:

1. KWIC filter design
2. Influence of the Nyquist factor
3. Sequence parameters and acquisition times
4. Results of *in vivo* $T_{1\rho}$ mapping in mice

Files:

- Online Figure 1, KWIC filter design
- Online Figure 2, Variation of the Nyquist factor, low values
- Online Figure 3, Variation of the Nyquist factor, high values
- Online Figure 4, Variation of the Nyquist factor, $T_{1\rho}$ maps
- Online Figure 5, Variation of the Nyquist factor, accuracy of $T_{1\rho}$
- Online Table 1, Sequence parameters and acquisition times
- Online Table 2, Results of in vivo $T_{1\rho}$ mapping in mice

1. KWIC filter design

For the design of the KWIC filter geometry the Nyquist criterion was considered. For each segment (containing n_ϕ projections, Fibonacci numbers) a maximum number n_r of samples in k_r direction was calculated, which defines the radii of the KWIC filter. In order to meet the Nyquist criterion, the following relation must apply:

$$n_o \geq 2\pi\, n_r \qquad n_o = 2\, n_\phi \qquad n_r \leq \frac{1}{\pi}\, n_\phi$$

Here, $n_o = 2\, n_\phi$ is the number of samples in angular direction. In order to definitely meet the Nyquist criterion in all regions of the segments and to prevent rounding errors in the implementation, an additional Nyquist factor f_{nyq} was added to the condition and floor functions were used.

$$n_r \leq \left\lfloor \frac{1}{\pi \cdot f_{nyq}}\, n_\phi \right\rfloor$$

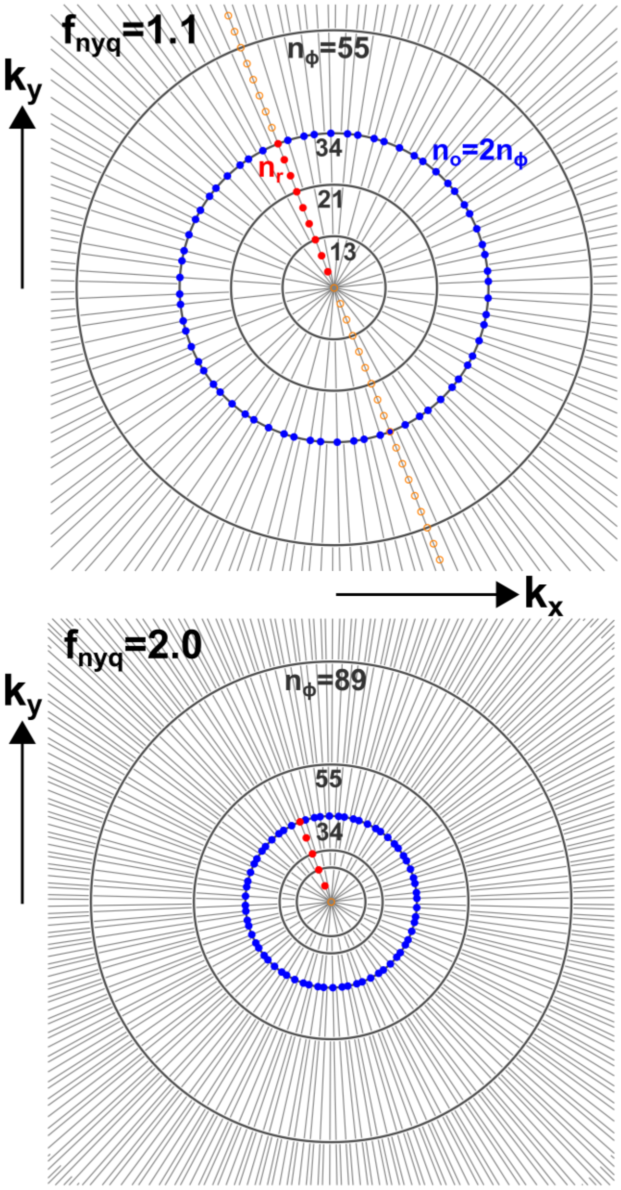

	f_{nyq} = 1.1	f_{nyq} = 2.0
n_ϕ	n_r	
**13**	3	2
**21**	6	3
**34**	9	5
**55**	15	8
**89**	25	14
**144**	41	22
**233**	67	37
**377**	109	60

Online Figure 1. Design of the KWIC filter and explanation of n_o, n_r and n_ϕ for two exemplary Nyquist factors f_{nyq}.

2. Influence of the Nyquist factor

The following section examines the influence of the KWIC filter design on the image quality and the $T_{1\rho}$ quantification. For this purpose, the measurements of the phantom study (BSA, 4 concentrations, t_{SL}=4…102ms, f_{SL}=1500Hz, Bloch Sorting) were considered and images/maps were reconstructed using different Nyquist factors. The $T_{1\rho}$ quantification accuracy was finally compared with the TSE reference for the different Nyquist factors.

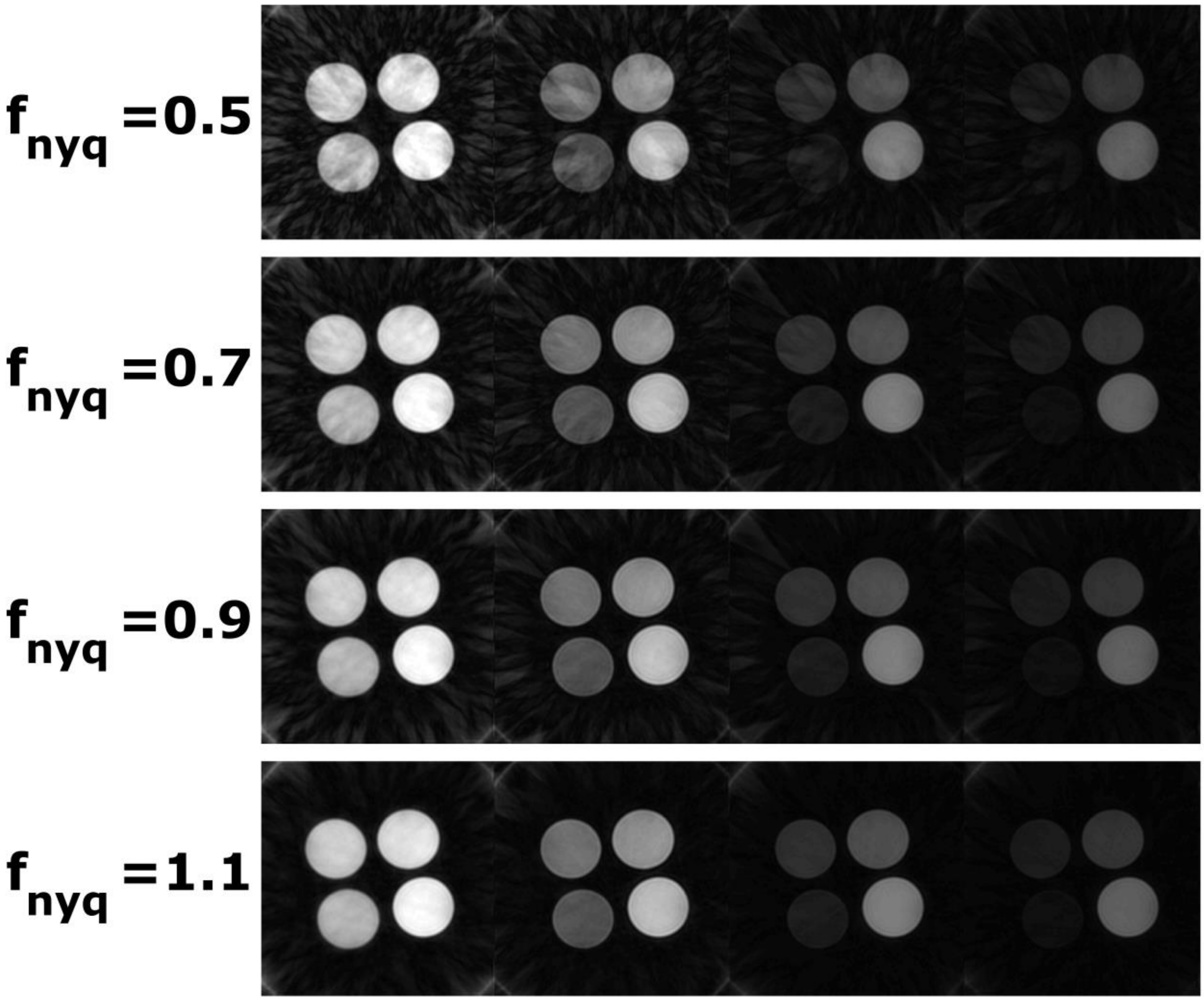

Online Figure 2. Variation of the Nyquist factor f_{nyq}, low values. Reconstructed images for different f_{nyq} (top to bottom) and different spin-lock times t_{SL} (4,32,74,102ms left to right). For the lowest factors, undersampling artifacts are clearly evident in all images and the SNR is reduced. For values f_{nyq}≈1, these artifacts decrease and are limited to background noise and slight streaking.

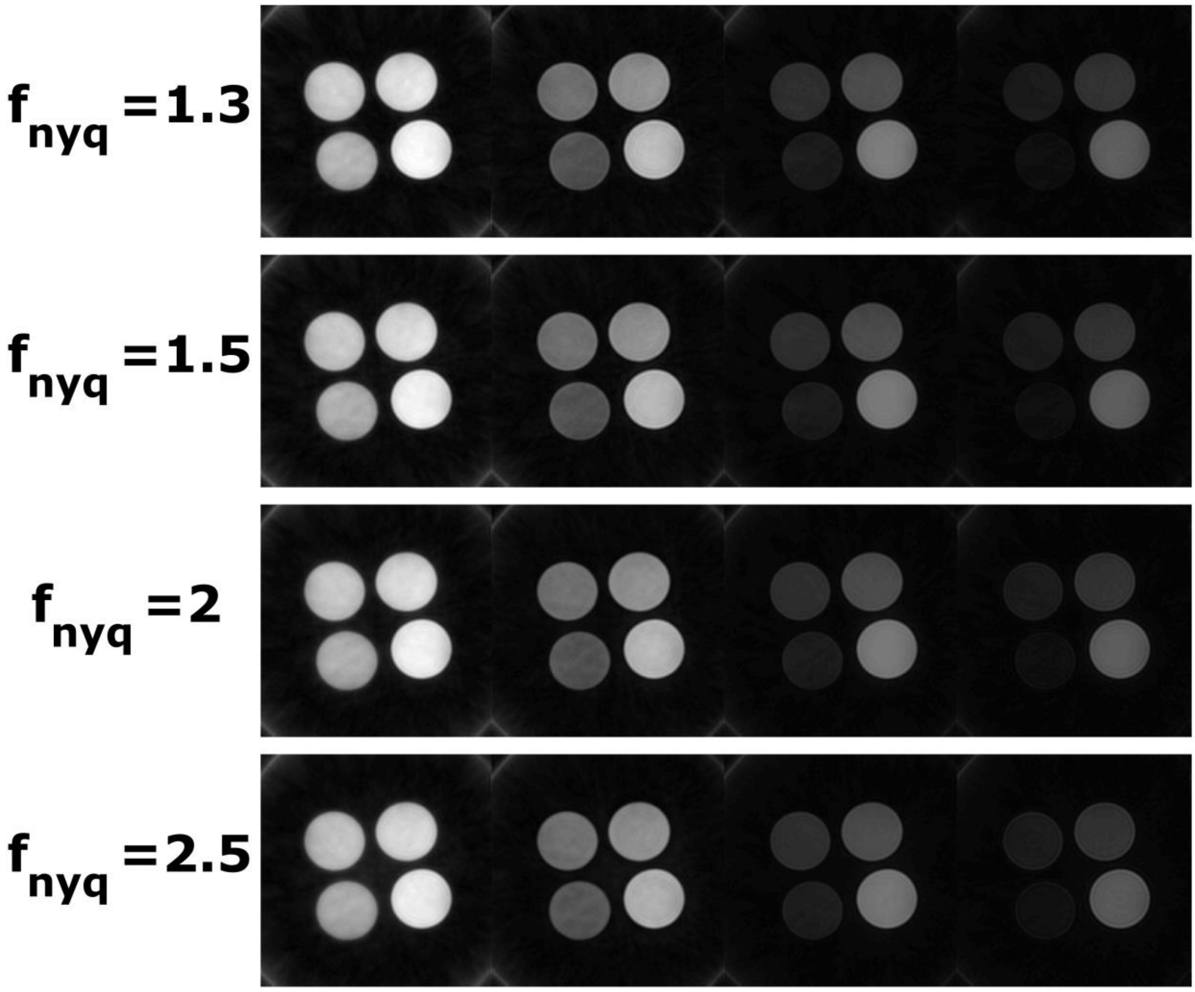

Online Figure 3. Variation of the Nyquist factor f_{nyq}, high values. Reconstructed images for different f_{nyq} (top to bottom) and different spin-lock times t_{SL} (4,32,74,102ms left to right). With increasing factors, it can be seen that low t_{SL} values show edge blurring. For high t_{SL} values edge sharpening can be seen. This effect was also discussed in [31]. Thus, the image quality cannot be increased by further rising the Nyquist factor.

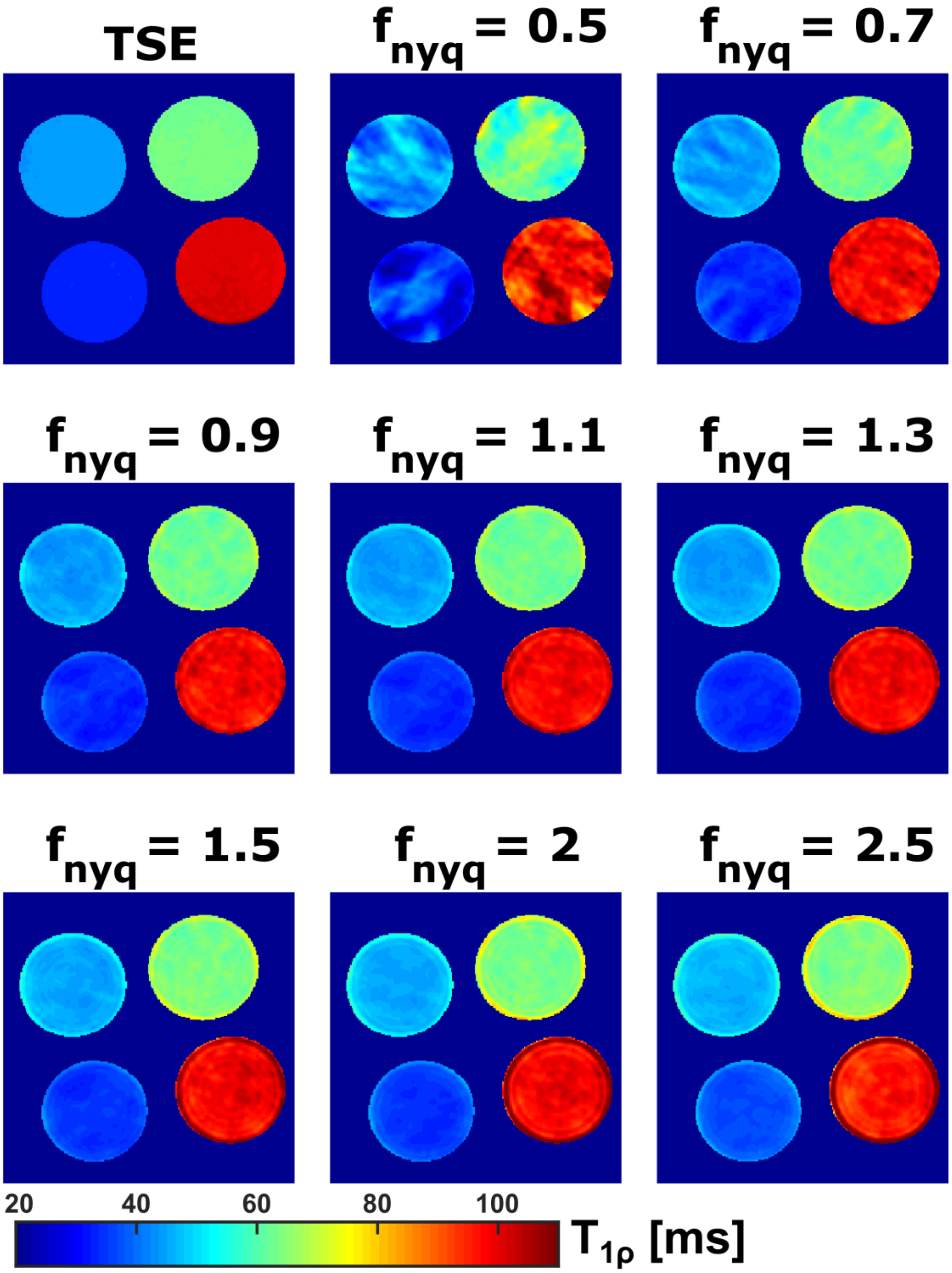

Online Figure 4. $T_{1\rho}$ maps for different Nyquist factors f_{nyq}. Compared to the TSE reference, the maps with moderate Nyquist factors between 1.1 and 1.3 show the best image quality. With lower factors, streaks occur within the phantoms and with higher factors, edge sharpening occurs.

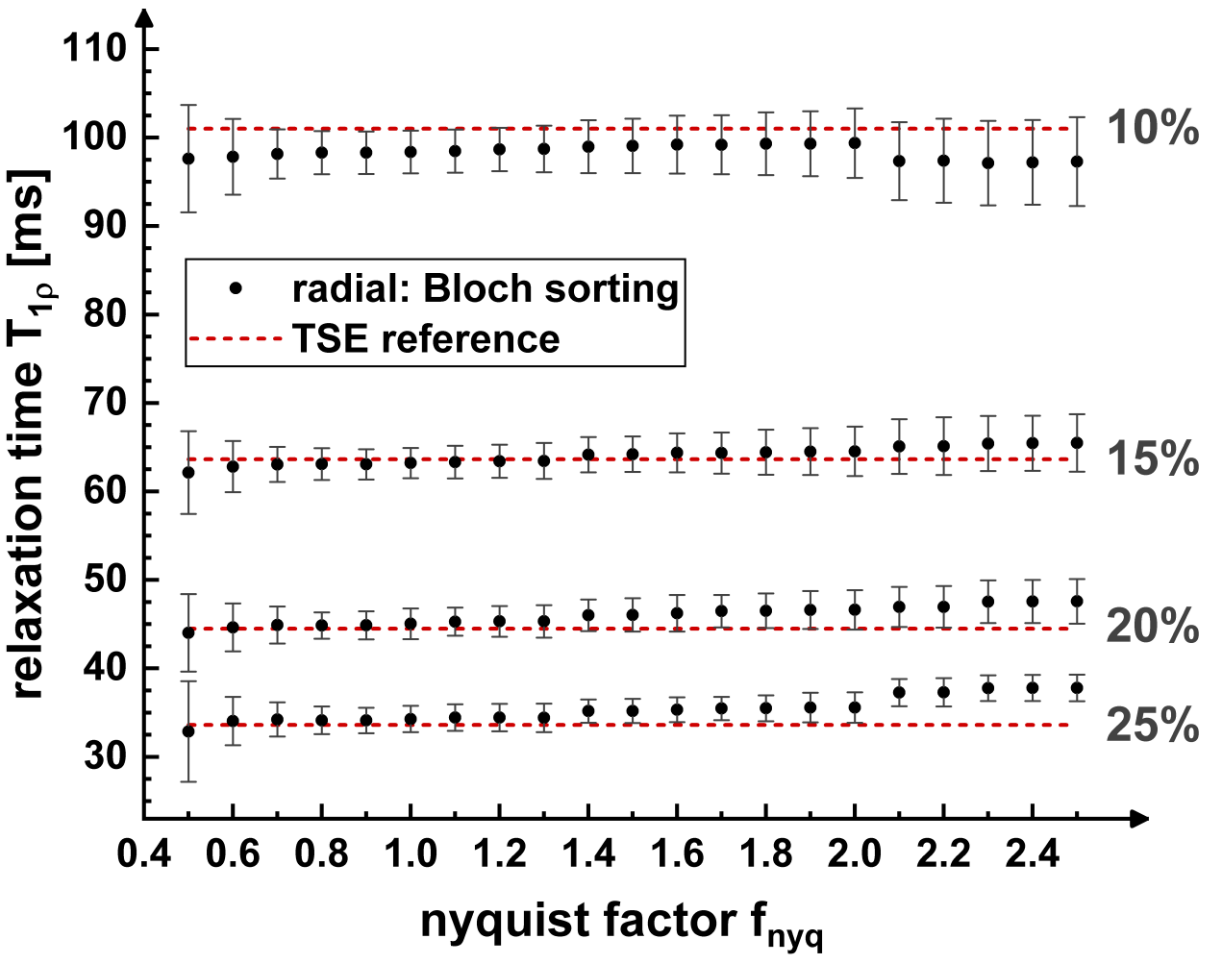

Online Figure 5. $T_{1\rho}$ quantification accuracy for different Nyquist factors f_{nyq}. The accuracy was determined in comparison to the TSE reference. Low factors (<1.0) result in large fluctuations in $T_{1\rho}$ within the phantoms due to the streaking artifacts and low SNR. Moderate Nyquist factors (1.0…1.3) show a high level of accuracy and only provide systematic errors for the phantom with the highest $T_{1\rho}$ relaxation time (BSA concentration 10%). High factors (>1.3) show systematic errors in all phantoms. Here, it can be seen that phantoms with low $T_{1\rho}$ tend to systematically higher values and vice versa. This is the result of an excessive mix of contrasts by the KWIC filter. The evaluation shows that moderate Nyquist factors in the range ≈1.1 are recommended for high quantification accuracy, which were used in the present work.

3. Sequence parameters and acquisition times

	phantom study	
	**GRE rad**	**TSE cart**
**TE**	2ms	7ms
**TR**	5ms	31.4ms
**BW**	75kHz	50kHz
**α**	40°	90°, 180°
**fov**	38.4x38.4 mm^2	38.4x38.4 mm^2
**matrix**	128x128	128x128
t_{SL}	4…102ms x8	4…102ms x8
f_{SL}	750…2500Hz x8	750…2500Hz x8
**NR**	4	4
N_{prep}	13	32
t_{rec}	5000ms	5000ms
t_{acq} **(1x map)**	8.7min	21.3min
t_{acq} **(dispersion)**	69.3min	170.7min
$T_{1\rho,BlochSorting}$	60ms	---
$T_{1,BlochSorting}$	1400ms	---

	in vivo **study**	
	**GRE rad**	**GRE cart**
**TE**	1.9ms	1.9ms
**TR**	4.7ms	4.7ms
**BW**	75kHz	75kHz
**α**	40°	40°
**fov**	32x32 mm^2	32x32 mm^2
**matrix**	128x128	128x128
t_{SL}	4…60ms x8	4…60ms x8
f_{SL}	750…2500Hz x8	1500Hz
**NR**	4	4
N_{prep}	13	32
t_{rec}	≈1460ms	≈1460ms
t_{acq} **(1x map)**	2.5min	6.2min
t_{acq} **(dispersion)**	20.2min	---
$T_{1\rho,BlochSorting}$	40ms	---
$T_{1,BlochSorting}$	1400ms	---

Online Table 1. Sequence parameters and acquisition times used for the phantom and *in vivo* study. The total acquisition times in the in vivo experiments depend on the respective respiratory cycle rates. These can be found in the physiological data in the Online Table 2.

4. Results of *in vivo* $T_{1\rho}$ mapping in mice

animal	relaxation time $T_{1\rho}$ [ms] LV	AHA 1	AHA 2	AHA 3	AHA 4	AHA 5	AHA 6	R^2 [0…1]	breath [ms]	ecg [ms]
1, I	39.3±3.0	36.5±1.6	39.1±2.3	42.2±2.4	41.4±2.8	39.6±2.4	38.2±2.9	0.996	1574±89	145.6±2.0
1, II	39.1±2.9	36.7±1.7	38.3±2.1	42.8±1.5	40.5±2.3	39.3±2.2	38.5±3.5	0.996	1610±79	145.8±1.8
1, III	39.0±2.9	36.4±1.7	38.6±2.2	41.0±2.5	40.5±2.7	39.1±2.0	39.3±3.8	0.997	1616±74	145.3±1.6
1, IV	38.1±2.7	36.6±1.4	38.6±2.3	40.4±3.7	37.7±2.9	38.5±1.7	37.1±2.4	0.997	1549±81	143.9±2.2
1, V	38.3±2.4	36.1±1.5	38.9±1.7	39.1±2.6	38.9±2.2	38.7±1.7	38.0±3.0	0.997	1479±72	143.1±2.5
1, VI	38.0±2.8	36.0±2.1	38.4±1.9	39.6±2.5	38.3±2.9	37.8±1.6	38.8±4.3	0.996	1408±76	142.0±1.8
1, VII	38.2±2.9	35.6±2.0	38.4±2.1	38.3±2.6	39.5±2.8	38.8±1.7	39.1±4.2	0.996	1383±73	141.5±1.8
1, VIII	37.7±2.8	36.0±2.1	37.9±1.8	37.9±3.4	38.2±2.7	38.2±2.1	38.4±3.9	0.996	1385±64	141.6±1.4
1, IX	38.9±3.0	36.3±1.5	39.2±2.3	40.1±3.4	41.0±2.4	38.8±2.1	38.4±4.0	0.995	1369±66	140.7±1.8
1, X	38.5±2.7	38.1±2.5	38.8±2.5	38.0±2.3	39.6±2.0	38.1±1.9	38.7±4.1	0.997	1341±70	140.3±1.7
**1, mean**	**38.52±0.54**	**36.43±0.68**	**38.62±0.39**	**39.94±1.70**	**39.55±1.27**	**38.70±0.56**	**38.44±0.62**	**0.996**	**1471±107**	**143.0±2.1**
2, rad	38.3±3.3	36.3±1.6	35.1±1.5	38.8±2.8	38.5±2.0	40.5±2.5	41.9±3.5	0.988	1412±73	131.5±1.2
2, cart	37.8±3.7	36.0±2.5	35.5±2.6	38.8±3.8	38.6±3.5	38.8±3.4	39.0±4.1	0.991	1757±75	153.2±0.7
3	37.9±3.7	36.0±2.0	35.7±1.8	43.3±2.3	41.6±3.4	36.4±2.9	37.1±2.3	0.992	1402±66	139.7±2.7
4	40.5±2.6	38.5±1.8	39.9±1.9	40.2±2.7	42.4±2.1	41.8±2.4	40.6±2.6	0.996	1198±62	131.2±4.2
5	41.8±4.0	41.0±2.2	37.9±1.9	43.4±3.2	45.3±5.2	40.1±2.1	45.3±1.8	0.990	1435±73	136.4±5.1
6	39.4±3.2	38.1±1.9	39.2±1.7	43.9±2.2	39.1±2.9	36.6±2.2	40.6±2.6	0.995	1631±52	147.4±1.3
7	39.2±2.5	37.6±2.4	39.6±1.9	42.0±1.5	38.9±2.4	38.0±1.1	39.1±2.4	0.992	1522±85	135.1±4.7
8	40.4±3.3	38.0±2.5	40.6±3.4	42.0±3.6	42.4±2.6	41.4±1.7	38.4±2.7	0.994	1604±80	130.9±4.1
9	40.0±3.6	36.6±3.0	39.7±2.4	46.0±2.7	42.5±2.5	40.2±2.6	39.8±1.7	0.990	1542±81	137.5±11.1
10	39.1±2.5	38.3±1.4	39.6±1.7	37.8±2.5	41.0±2.5	41.1±2.4	37.2±2.4	0.995	1206±47	129.1±2.7
**mean**	**39.5±1.2**	**37.7±1.5**	**38.6±1.8**	**41.7±2.5**	**41.1±2.1**	**39.4±1.9**	**39.7±2.4**	**0.993**	**1460±154**	**137.3±5.9**

Online Table 2. $T_{1\rho}$ quantification results in N=10 different animals for f_{SL}=1500Hz. Animal 1 underwent n=10 identical measurements in direct succession and in animal 2 a fully sampled cartesian reference measurement was carried out. The table also shows the results in the individual AHA segments for $T_{1\rho}$, as well as the mean R^2 values in the global left ventricular ROI (LV) and the monitored physiological parameters (cardiac cycle and breath cycle length).
